# Limitations of Cluster-Trained MLIPs for Liquid Density and Diffusivity

**DOI:** 10.1021/acs.jctc.5c02043

**Published:** 2026-03-31

**Authors:** Viktor Svahn, Ioan-Bogdan Magdău, Samuel P. Niblett, Gábor Csányi, Kersti Hermansson, Jolla Kullgren

**Affiliations:** † Department of Chemistry-Ångström, 8097 Uppsala University, Box 538, S-75231 Uppsala, Sweden; ‡ School of Natural and Environmental Sciences, 98458 Newcastle University, Newcastle Upon Tyne NE1 7RU, U.K.; § Yusuf Hamied Department of Chemistry, 2152 University of Cambridge, Lensfield Road, Cambridge CB2 1EW, U.K.; ∥ Dassault Systèmes BIOVIA, 334 Cambridge Science Park, Cambridge CB4 0WN, U.K.; ⊥ Engineering Laboratory, University of Cambridge, Cambridge CB2 1PZ, U.K.

## Abstract

Machine-learned interatomic potentials (MLIPs) based on quantum-mechanical data are often used as a means to combine the performance of classical force-fields with the accuracy of electronic structure methods. In this work, MLIPs based on the MACE architecture were trained, starting from two publicly available data sets: one based on periodic structures, and the other based on molecular cluster data. Two rather challenging liquid properties are in focus, density and diffusivity, here for the battery-relevant ethylene carbonate and ethyl methyl carbonate solvents and mixtures thereof. The focus of our study is the uncertainties in the generated MLIP models themselves (calculated for committees of models with different regression seeds and different training set sizes) and how these uncertainties reflect on the MD-simulated target properties. The second focus point is whether these uncertainties are small enough to allow the comparison and assessment of different density functional theory (DFT) functionals; here, only a small number of them are compared, but the workflow opens up for a more extensive assessment of many DFT functionals. We find that all our MACE-MLIPs, both cluster-trained ones and the periodic-structure-trained ones, produce stable 1 ns NPT trajectories, regardless of training set size and cluster composition, but the MACE-MLIPs trained on cluster data (labeled with the hybrid ωB97X-D3 functional) are found to be sensitive to both the random training seed and the data selection, resulting in large uncertainties on the simulated diffusivity and density values.

## Graphical abstract

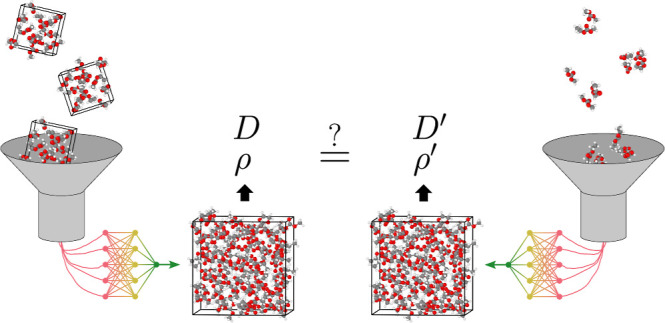

## 1 Introduction

Density and diffusivity are two key properties of molecular liquids. They are related via the strengths of intermolecular interactions and can both be calculated from MD simulations, where the system density generally considerably easier to converge than diffusivity. The latter typically requires long-time simulations in large simulation boxes, and, during many decades, the use of classical force fields has proven indispensable in achieving this task. Even so, the accuracy of inter- and intramolecular interactions obtained from classical force fields is—in principle, and most often in practice as well—limited compared to that of quantum-mechanical electronic structure methods such as density functional theory (DFT).^1–3^ Currently, the development of DFT-based machine-learned force fields has opened up the possibility to predict properties with DFT accuracy using MD simulations. Here, as a proof of concept, we will make use of such a strategy to compare three DFT functionals in terms of their abilities to describe the density and diffusivity of battery-relevant ethylene carbonate (EC) and ethyl methyl carbonate (EMC) solvent systems. All these things considered, the path toward achieving stable machine-learned force fields, or interatomic potentials (Machine-learned interatomic potentials (MLIPs)), with DFT quality is unfortunately not always a straight one. MLIPs can be generated (fitted) offline, followed by running MD simulations or, alternatively, by fitting energies and forces on-the-fly, where the MLIP is gradually improved as the dynamics progresses and the model accumulates more data. Both these strategies have been used in the literature to enable molecular simulations of extended length and time scales at an accuracy that approaches the DFT world.^4–7^ Multiple rounds of active learning is typically required before an MLIP becomes stable enough to be used for MD production (see, for example, the work by Magdau et al.^8^). Generating the necessary training sets for MLIPs can therefore be both time-consuming and highly resource intensive. A way around this challenge is to either rely on publicly accessible databases^9,10^ or reuse published training sets that have been demonstrated to produce reliable MLIPs. Moreover, there are many different flavors of MLIPs to choose from^4–7,11,12^ including the equivariant message passing graph neural network, MACE.^12^ Niblett et al. successfully trained MACE models on a data set^8^ that had been generated from active learning using a different MLIP architecture and showed that MACE-MLIPs demonstrate impressive stability across a committee of trained MLIPs without the need for data augmentation.^13^

Another challenge is the selection of an appropriate electronic structure method for the system and purpose at hand. The intermolecular interactions in EC:EMC systems are dominated by electrostatic dipole–dipole interactions (permanent dipoles and weaker induced ones) as well as dispersion interactions. Thus, in addition to the exchange–correlation treatment in DFT calculations, the dispersion interaction treatment needs attention. DFT functionals are sometimes classified according to Perdew’s “Jacob’s ladder of DFT functionals.”^14^ In this hierarchical picture, climbing Jacob’s ladder can be viewed as a systematic path toward higher accuracy, albeit at the price of increased computational effort. A large number of benchmark studies in the literature assess the capabilities of DFT functionals, sometimes even hundreds of them.^15^

In the first part of this study, we assess the impact of the DFT functional on the MLIP models and, more specifically, on the MD-generated diffusion and density values of our target systems. A modest set of periodic training structures (960 of them, prepared in our previous work^8^) are used. These structures are here labeled using three DFT functionals, PBE-D2, PBE-D3, and B97-D3, selected because Perdew, Burke, and Ernzerhof (PBE) and B97 are much used in the literature and because they exhibit some interesting similarities and differences. A key question in this study is whether these MACE-based MLIP models, trained on periodic data, manage to yield liquid densities and diffusivities of sufficient precision to discriminate between the selected DFT methods. Our answer will be “yes, they manage,” which we will subsequently contrast with results from models trained on DFT cluster data, using the same regression and MD simulation strategies.

The second part of this work thus deals with MLIPs trained on cluster data. With the emerging methodologies for fine-tuning foundation models,^16–18^ a wider use of MLIPs based on advanced electronic structure methods such as hybrid-DFT methods or, say, coupled-cluster methods, may soon become reality. Here, cluster-based MLIPs may appear as a natural choice. There are already successful examples of such a strategy, see, for example, the study of fine-tuned MLIPs for Ice Ih by Gawkowski et al.^19^

Foundation MLIP models can demonstrate stable dynamics off-the-shelf,^19–21^ but they still require additional fine-tuning to converge thermodynamic and kinetic properties. This is highlighted by the recent work of Beiersdorfer et al.^22^ who compared a Gaussian Approximation Potential (GAP) and MACE to the MACE-MP-0 ^20^ foundation model, fine-tuned to a liquid similar to the ones studied here, but with ions included. They found that MACE-MLIPs trained directly on their data set outperformed both GAP and MACE-MP-0. Our own GAP versus MACE comparison using the periodic data set show a similar behavior (see Figure S3 in the SI).

In two recent studies, cluster-based data were used to create MLIPs for EC, EMC, and other solvents in combination with ions.^23,24^ Also, in our current study, we have generated MLIPs based on higher-level (hybrid) cluster-based DFT data, here with the approach to use exactly the same MACE regression scheme and settings as those for the periodic training data in the first part of this study. Also, in this part of the paper, the purpose is to examine whether the resulting MLIPs (now cluster-based) will yield diffusivity and density values of a similar precision as those resulting from the periodic-data-based MLIPs; this would then allow us to assess such higher-level DFT functionals and wave function-based methods on the same footing as the three functionals.

In the context of condensed-matter simulations, the use of cluster training data entails certain new challenges compared to periodic training data, in particular regarding the risk of not using (a sufficient number of) bulk-representative structures in the data set. Problems with cluster-to-liquid extrapolation and out-of-domain transfer may potentially result.

In going from cluster-based MLIPs to bulk applications, the out-of-domain effect has at least two conceptually different origins. First, atoms that reside in the outer parts of a cluster do not provide examples of atomic environments that are representative of the liquid phase. Only atoms that are “rather well embedded” in a cluster provide such examples. Second, the use of finite clusters is an effective truncation and omission of long-range effects within the training data itself, both direct long-range effects and nonadditive cooperative effects such as polarization; in short, the training set will contain skewed forces and energies compared to typical bulk-like environments. The potential problem with clusters therefore becomes 2-fold: (i) Are there enough representations of atomic environments with local packing similar to a liquid? (ii) Will the exclusion of long-range effects lead to systematic errors in the generated MLIPs (subsequently in the simulated system properties)?

In the first case, poor representation of liquid-like local environments might force models to extrapolate when used in simulating liquid systems, and in the second case, the training-set forces and energies may be nonrepresentative, as they originate from skewed electron density distributions. Reference 25 provides indications that the training of neural networks on out-of-domain data may lead to considerable extrapolation errors that are sensitive to the random seed used during model initialization which motivated Gong et al.^24^ to consider possible variations due to random seeds. In particular, they observed committee errors of the same order as in the work by Niblett et al. (0.03 g cm^–3^ for the densities of EC and EMC). While Gong et al. initially started their training from the data set generated by Dajnowicz et al.,^23^ they required a few rounds of active learning until stable MLIPs were obtained.

We examine whether the stability and reproducibility that characterize our MACE-MLIPs generated from periodic training data also hold for cluster-based training data.

Concerning stability, the answer is yes: all our MLIPs turn out to be capable of sustaining 1 ns long trajectories even when trained on modest amounts of the original data from ref 23. However, we simultaneously find a low agreement across a committee of predictions for the cluster-based MLIPs (much lower compared to that of refs 13 and 24), which then also affects the capability of the MD simulations to discriminate between results from different DFT methods. Overall, the predictions from our cluster-based MLIPs are found to be highly sensitive to data selections, highlighting the problems of out-of-domain training.

The layout of the paper is as follows. The Method section presents, in order, the data sets used for MLIP training, the DFT functionals and DFT calculations, the MLIP generation and adhering regression quality, and, finally, the MD simulation details and the MD property analyses. In the Results and Discussion section, we discuss our workflow for the systematic comparison of the MLIP-driven MD simulation results, especially with respect to system densities and molecular diffusion coefficients, and with special focus on the (possible) differences observed depending on the choice of DFT functional for the training-set data. The first part (Section 3.1) presents the MD results from MLIPs constructed from periodic training data, while the second part (Section 3.2) presents results from MLIPs based on training data consisting of molecular clusters. In Section 3.2, in addition to the DFT functionals, also data set size, data set sample, and variations in model prediction due to the training seed become important items of comparison and attention.

## 2 Methods

### 2.1 Data Sets for MLIP Generation

The structures for the generation of our MLIPs were taken from two sources: periodic structures from the work of Magdău et al.^8^ and cluster structures from the work of Dajnowicz et al.^23^ The respective publications describe in detail how their training sets were developed, involving many rounds of active learning with systematic potential energy scans, as well as MD simulation snapshots. In the current study, we make use of these training structures as the basis for our MLIPs. The data sets are described in Table 1 together with information regarding the number of structures and atoms present in them.

**Table 1:** Summary of Data sets Used in this Work. The Table Lists Data set Names, Data set Sizes in Terms of Number of Structures and Atoms, and Provides a Short Description of the Contents of Each Dataset

data set	number of structures	number of atoms	structure types
Periodic	935	66,188	MD snapshots, monomers, and volume scans
Clusters-Small	8016	327,089	monomers and gas-phase clusters (*N*_mols_ ≤ 6)
Clusters-Medium	42,824	1,269,916	monomers and gas-phase clusters (*N*_mols_ ≤ 6)
Clusters-Large	221,824	4,805,916	monomers and gas-phase clusters (*N*_mols_ ≤ 6)
Clusters-Full ^a^	362,382	7,781,985	monomers, gas-phase clusters (*N*_mols_ ≤ 6), ions and non-singlet electronic states

a Not used by us for training models.

Volume scans were included in the periodic data set that we use; the volume scans explicitly sample variations in density and the associated virial pressure response. Concerning cluster-based training sets, it is worth noting that the training set of Dajnowicz et al., which we make use of, actually contains clusters of varying “densities” in the sense that there is a large variation in the compactness of the clusters (see Figures 1 and S2 in the SI). In this way, the cluster data in fact indirectly contain information akin to that captured by volume scans. However, information about virial pressure is missing in the cluster data.

**Figure 1:**
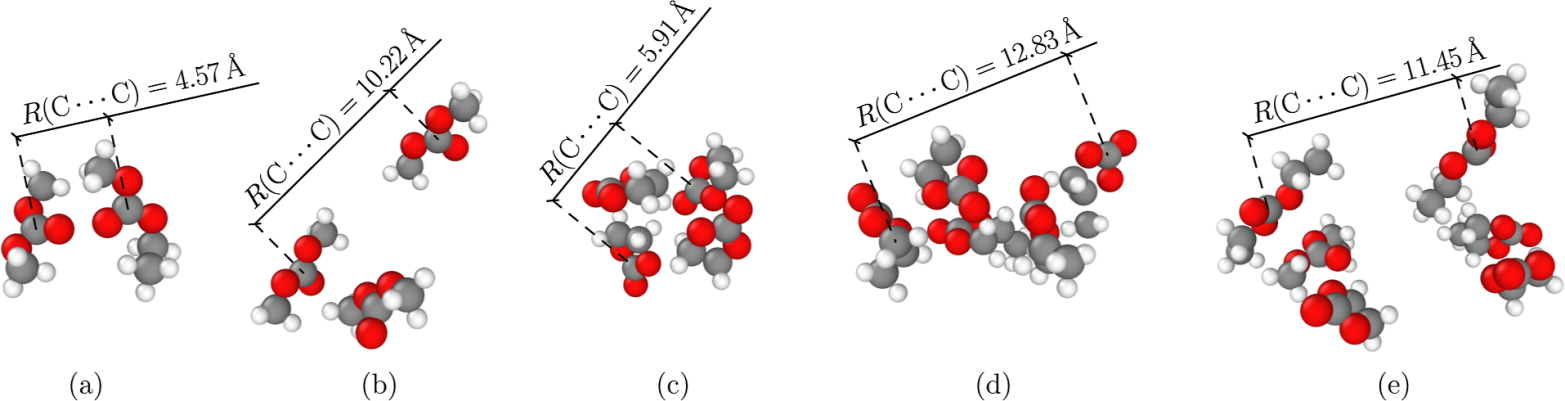
Different examples of clusters, ranging from dimers to hexamers (a–e), that are part of the Clusters-Large data set. These images illustrate the varied compactness of clusters in the data set.

We denote the data set of periodic structures Periodic and the full data set of the cluster structures Clusters-Full. The data sets contain geometries, virials (only the Periodic data), energies, and forces. We created a data set, denoted Clusters-Large, by selecting from the Clusters-Full data set all charge-neutral clusters with spin multiplicity 1, containing EC, PC, VC, DMC, EMC, or DEC molecules. We then created additional data sets from the Clusters-Large data set by selecting 25% (Clusters-Medium), or 6.25% (Clusters-Small), of the structures from it in such a way that as many of the large clusters as possible were retained to minimize the oversampling of specific compositions, such as pure EC. Some statistics related to these data sets is listed in Table 1. The DFT labelling of the structures is treated in the next section and details of the MLIP generation are described in the section after that. A list of all MLIPs trained in this work is given in Table 2.

Three variants of the Clusters-Medium data set were created, sharing the same distribution of molecular composition but consisting of different specific data points (see Figure 2). Similarly, three data set variants of the Clusters-Small data set were created.

**Figure 2:**
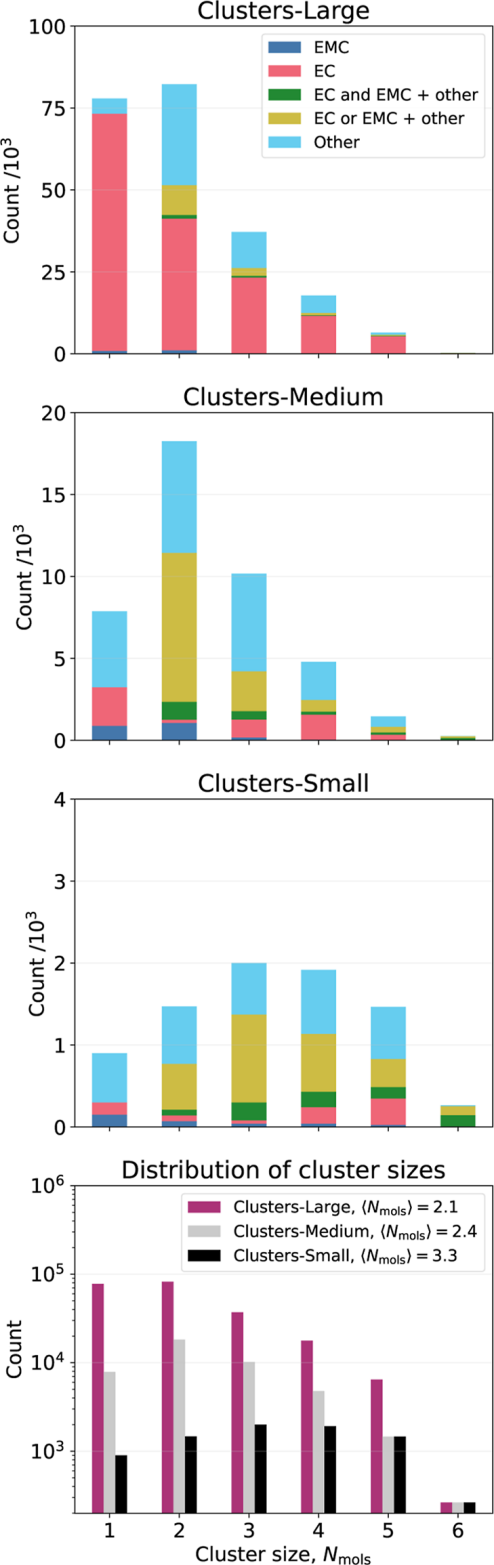
The first three panels show the distribution of compositions over the differently sized clusters for the three data set sizes. In these diagrams, green indicates structures that contain both EC and EMC and possibly something else (other), whereas yellow color indicates the presence of either EC or EMC and possibly something else (other). The term “other” refers to propylene carbonate (PC), vinylene carbonate (VC), fluoroethylene carbonate (FEC), dimethyl carbonate (DMC), and diethyl carbonate (DEC). The bottom panel includes all three data set sizes and compares their cluster size distributions and average cluster sizes (Note the log-scale on the y-axis in this plot).

**Table 2:** Summary of MLIPs Trained in This Work; the Names are Shown as Dataset-*n*/Functional, Where *n* Denotes the Numbering of the Dataset Subsample; the Subsamples have the Same Compositions (namely those depicted in Figure 2) but Differ in Terms of Specific Data Points (See Text); the Number of Training Seed-Replicas of Each Model Is Shown as Well

training set	number of seeds
Periodic/PBE-D3	1
Periodic/PBE-D2	1
Periodic/B97-D3	1
Clusters-Small-1/ωB97X-D3	3
Clusters-Small-2/ωB97X-D3	1
Clusters-Small-3/ωB97X-D3	1
Clusters-Medium-1/ωB97X-D3	3
Clusters-Medium-2/ωB97X-D3	1
Clusters-Medium-3/ωB97X-D3	1
Clusters-Large/ωB97X-D3	3
Clusters-Small-1/B97-D3	1
Clusters-Small-2/B97-D3	1
Clusters-Small-3/B97-D3	1
Clusters-Medium-1/B97-D3	1
Clusters-Medium-2/B97-D3	1
Clusters-Medium-3/B97-D3	1
total number of models	22

### 2.2 DFT Functionals

For the periodic systems, the DFT calculations were performed using a plane-wave basis set in conjunction with Projector Augmented Wave^26^ type pseudopotentials^26^ as implemented in the Vienna Ab initio Simulation Package.^27–30^ We used the PBE^31^ and B97 functionals^32^ with Grimme’s D3 corrections^33^ and Becke–Johnson (BJ) damping.^34^ The plane wave basis set was truncated using a kinetic energy cutoff of 800 eV. The Brillouin zone was sampled at the Γ-point only. We named these training sets Periodic/PBE-D3 and Periodic/B97-D3. The Periodic/PBE-D2 training set contains the same structures as the other periodic data sets but was labeled with energies and forces from CASTEP^35^ calculations with the same energy cutoff and *k*-point sampling as the other periodic training sets. The Periodic/PBE-D2 training set was taken as is from ref 8.

For the cluster MLIPs, the DFT labels in the original Clusters-Full data set by Dajnowicz et al.^23^ were computed by them with the Psi4 program,^36^ which uses local atomic Gaussian basis sets; they used the ωB97X-D3 functional^37^ with a Grimme D3­(BJ) dispersion correction and the def2-TZVP basis set^38^ with diffuse augmentation^39^ (def2-TZVPD). For clusters, no new DFT calculations were performed by us at the ωB97X-D3 level, as our Clusters-Large/ωB97X-D3, Clusters-Medium/ωB97X-D3, and Clusters-Small/ωB97X-D3 data sets (cf. Table 1) are all subsets selected by us from the Clusters-Full data set. However, we performed a set of cluster DFT calculations at the B97-D3­(BJ) level, also with Psi4 software and the def2-TZVPD basis set, for comparison with the ωB97X-D3­(BJ) results; those results are presented in Section 3.2. In the rest of this article, the “(BJ)” parenthesis is dropped from the D3 notation.

### 2.3 MLIP Generation

The machine-learning interatomic potentials were trained by using the MACE framework. The training data were taken from the data sets in Table 1, with 5% of the configurations reserved for validation. The reference data also included the atomic energies (E0s), computed at the corresponding DFT level for each training set.

The MACE architecture employed a message-passing equivariant neural network with two interaction layers, a correlation order of 3, angular momentum channels up to l = 3, and hidden irreducible representations of size 128 × 0e + 128 × 1o. The atomic environment in each message-passing layer with a smooth cutoff radius of 6.0 Å uses five basis functions.

A weighted loss function with contributions from energies (weight 1.0) and forces (weight 100.0) was minimized during the training, using equal weights across configuration types. Optimization was carried out with the Adam method (of the AMSgrad flavor) using a batch size of 20, a weight decay of 5 × 10^–7^, a learning rate of 0.01, and an exponential moving average (EMA decay 0.99). Models were initially set to train for 1600 epochs with early stopping (patience 50). In practice, models were considered finished when a plateau had been reached. The periodic models trained for around 750 epochs, whereas the number of epochs associated with the cluster data varied. Broadly, the small-cluster models used 1800 epochs, the medium models used 1200, and the large models used 400. Stochastic weight averaging was performed at the end of each fit to ensure low energy errors. Table S1 in the SI lists the training and validation RMSEs in energy per atom and forces for all MLIPs used in this study.

Models were fitted to the Periodic data set labeled with PBE-D2, PBE-D3, and B97-D3 energies, forces, and virial stresses; the results are shown in Figure 3. Overall, force RMSEs of the trained models compared to their labels were $O(10\,\;\mathrm{meV}\,\;{Å}^{-1})$ and the relative-RMSEs were less than one percent. The same figure also shows the error as a function of DFT reference force, revealing that the errors tend to be larger when the force is small. Most errors are very small (around 5 meV Å^–1^), but there is nevertheless a considerable number of data points where the error is substantial. To put this in perspective, in our simulations, an atom moves 0.025 Å per time step on average at temperatures around 300 K. Integration of the forces in Figure 3 over such a displacement yields a correlation that describes the training error of the forces in terms of an energy instead. Since this is a training error, this number gives a rough estimate of the lowest error per atom that may be associated with a single time step in an MD simulation and which can be viewed in relation to chemical accuracy (which is considered to be around 40 meV). In the present case, the resulting RMSEs (see Figure S1 in the SI) between integrated forces were less than 1 meV for all three models.

**Figure 3:**
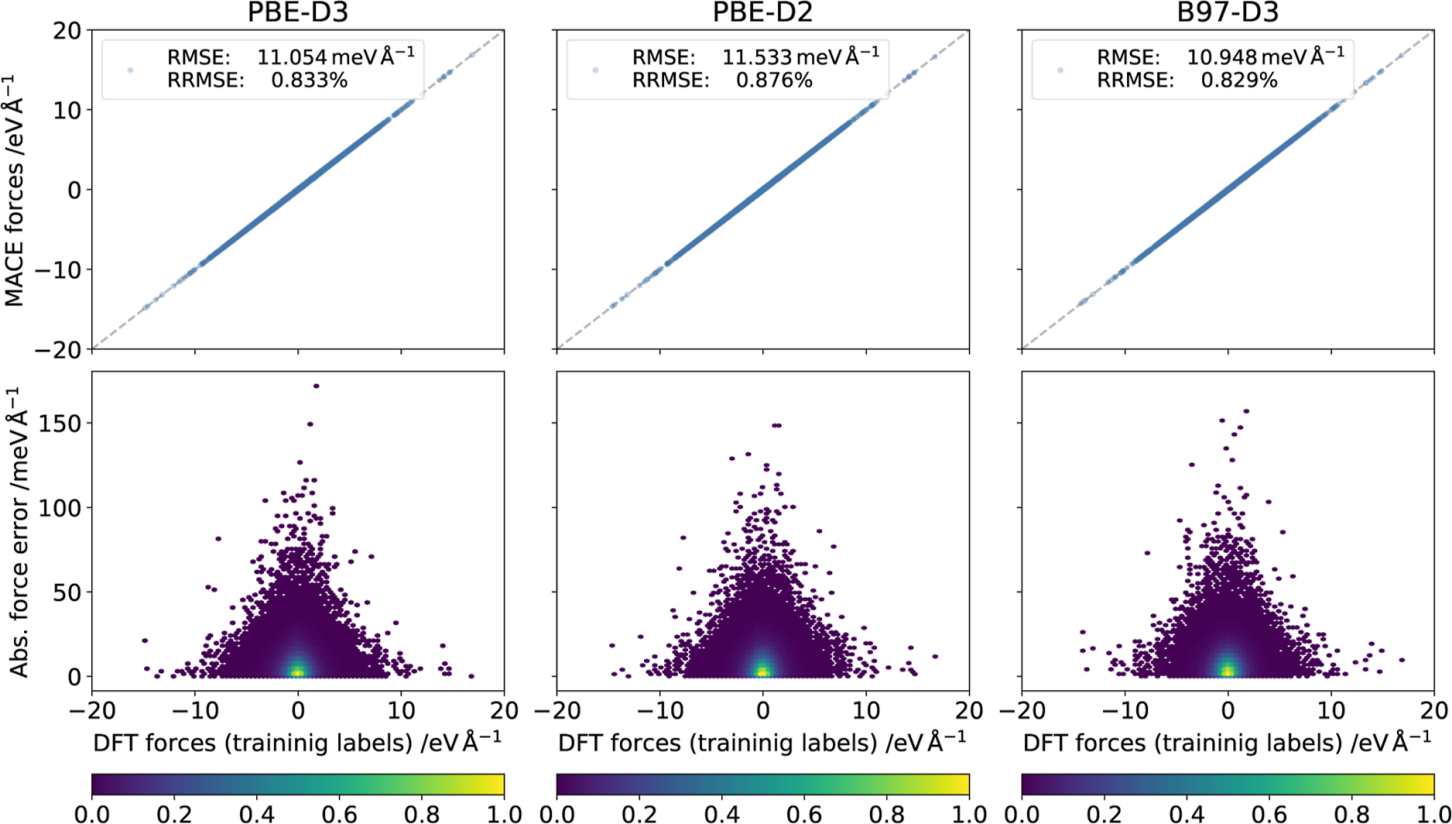
Effects of the DFT functional on the regression quality. Regression metrics (scatter plots, root-mean-squared errors, absolute force errors) are shown for model forces trained on the Periodic data set labeled with PBE-D2, PBE-D3, and B97-D3. The model forces are compared with their respective DFT labels. The color bar indicates the point density in each bin.

### 2.4 MD Simulations and Property Calculations

Four liquids with different compositions were studied: pure EC, pure EMC, and EC:EMC mixtures in 7:3 and 3:7 molar ratios. The initial (isotropic) box dimensions for the respective compositions were 22.54 Å, 22.09 Å, 22.30 Å, and 22.38 Å. With the exception of the 7:3 mixture, which consisted of 1005 atoms, all of the other liquids contained 1000 atoms.

The MD simulations were run in the *NPT* ensemble for a duration of 1 ns each, using our trained MLIPs. The simulations were performed under a constant pressure of 1 atm, and the target temperature was 298 K except for the pure EC system which was simulated at a slightly higher temperature of 313 K due to EC’s low melting point.

All MD simulations were performed using the MACE implementation (ver. 0.3.7) as a calculator in the Atomic Simulation Environment (ASE) package (ver. 3.22.1).^40^ A time step of 1 fs was used, and the simulations were performed with the Nosé–Hoover thermostat and the Parrinello–Rahman barostat with relaxation times of 50 and 2500 fs, respectively. The bulk modulus value that is required to specify the barostat in the ASE implementation was set to 2.0 GPa. The trajectories were initiated from MD snapshots taken from ref 8.

The diffusive regime is commonly said to have been reached once the logarithm of the time-dependent MSD curve has reached a unit slope with respect to log *t*, and we define *t*_*D*_ to be the time at which the diffusive regime has been reached.^41^ Since this regime is only transient between the ballistic and hydrodynamic regime, we calculated the log–log slopes over a moving window and selected the window with the best linear fit, with *t*_*D*_ being the starting time of said window. Here, we used a moving window of 2000 snapshots and allowed the slope to be 1.0. theory and computation.

Molecular diffusion coefficients were determined from the mean-squared displacements of the molecular center-of-mass by fitting the Einstein relation to the slopes of the MSD curves; they are reported with a 95% confidence level. The error bars in the graphs in Section 3.1 reflect this confidence. When diffusion coefficients are compared with the experiment, the former have been corrected for finite size effects using

1 $$D_{\infty }=D_{L}+\frac{2.837k_{B}T}{6\pi \eta L}$$

where *D*_*L*_ is our value obtained from the MSD slope, *k*_*B*_ is Boltzmann’s constant, *T* is the temperature, η is the dynamic viscosity of the liquid, and *L* is the average side length of the cubic simulation box. For more details regarding this expression, the reader is referred to refs 41 and 42.

## 3 Results and Discussion

### 3.1 Periodic Training Data: Impact of the DFT Functional on Densities and Diffusivities

The extrapolation capabilities of neural networks have been shown to vary, not only between specific implementations but also internally and with respect to the random seeds used in the training process.^25^ In the work of Niblett et al.,^13^ the effects of such seed variations on the simulated density and diffusivity of an EC:DMC liquid mixture were quantified for the very same MACE architecture that we have used in the present work. Compared to ref 13, in the present study, we deal with four solution compositions, and the scientific focus is different, but the results from ref 13 are helpful to us. In their work, across a committee of five MACE MLIPs, all trained on the very same data set but initiated with different seeds, the density of a single EC/EMC mixture at 500 K was found to vary by ± 0.02 g cm^–3^. Similarly, they found the error in the predicted diffusion coefficients across this committee to be around 1%. The motivation behind their use of elevated temperature was a desire to determine an upper bound to uncertainties in densities and diffusivities across the committee. Thus, as these modest committee errors with respect to the training seed were established for liquid EC:EMC in combination with MACE in ref 13, we have chosen to bypass such an assessment for our periodic-data-based MLIPs in the present study. In the following, we will therefore consider only a single model (one seed) trained for each periodic data set, i.e., for each DFT labeling of the Periodic data set.

Three DFT functionals were examined: PBE-D2, PBE-D3 and B97-D3. For a given functional, a comparison of the results for the different liquid compositions (red, yellow, green, blue in Figure 4) shows that the diffusivity more or less follows the inverse of the density; see also the Periodic results in Figure 5. Overall, the density of the Periodic/PBE-D3 model is found to be lower than those for the other two DFT labels. The differences in the average densities across compositions between the Periodic/PBE-D2 and Periodic/B97-D3 models is around 0.01 g cm^–3^, whereas the corresponding difference between Periodic/B97-D3 and Periodic/PBE-D3 is 0.1 g cm^–3^. Compared to committee averages from ref 13, the latter difference is about five times larger, i.e., significant.

**Figure 4:**
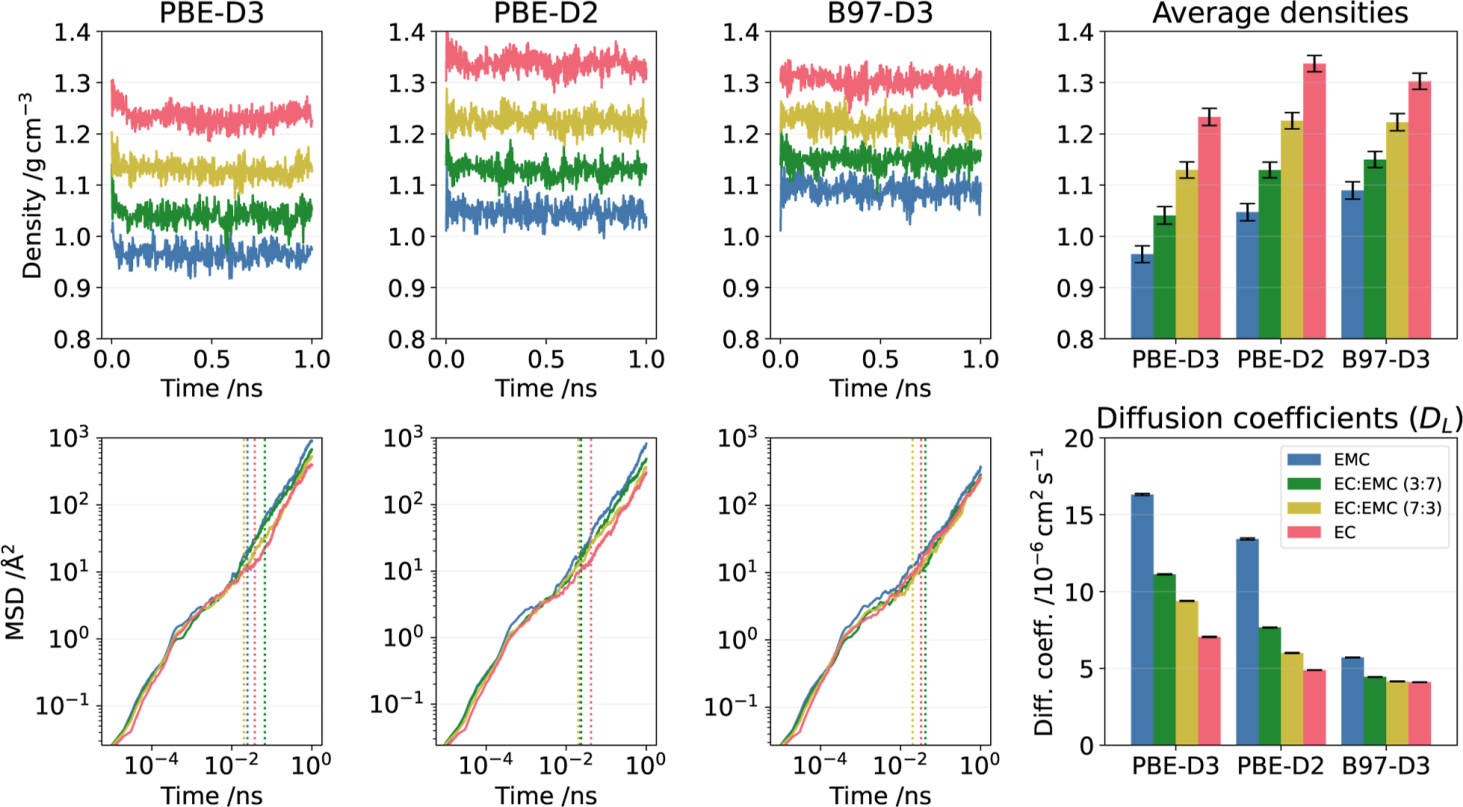
Influence of DFT functional on the MD-generated liquid properties targeted in this study. Results from MD simulations with three different MLIPs are shown, all trained on the Periodic data set structures labeled with either PBE-D3, PBE-D2, or B97-D3 values (energies, forces, stresses). The colors denote the liquid compositions in the MD simulations (cf. the legend in the south-east frame). The first three columns display the time evolution of the system densities (top) and of the mean-squared-displacements (MSD) (bottom) on a log–log scale. The bar diagrams in the rightmost column display the corresponding average density (top frame) and the diffusion coefficients (*D*_*L*_) (bottom frame) fitted from the MSD curves, starting from the respective vertical lines (*t*_*D*_) in the MSD time evolution graphs.

**Figure 5:**
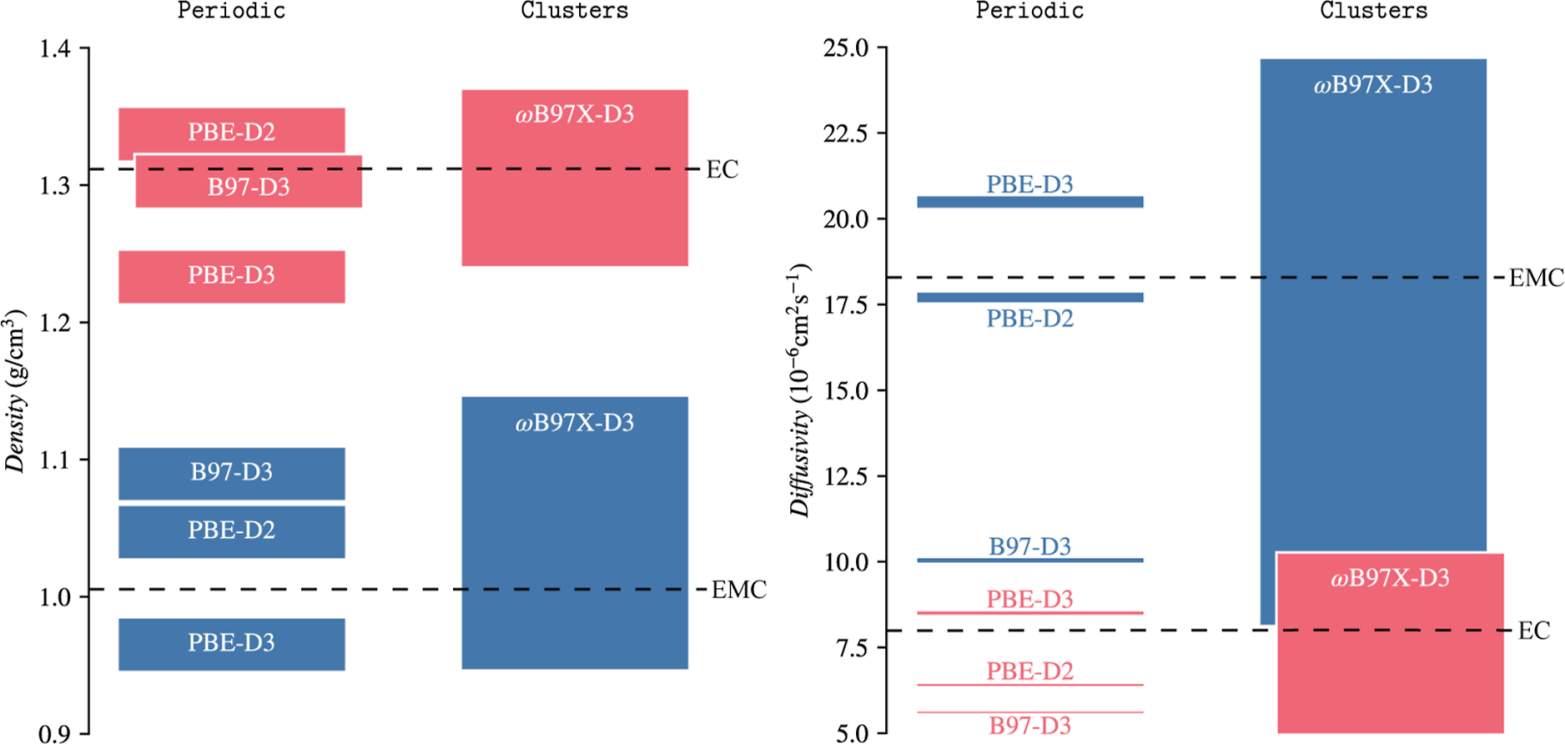
Comparison of MD-simulated densities and diffusion coefficients for pure bulk EC (red) and pure bulk EMC (blue). The MD simulations were performed with the MLIPs based on periodic or cluster training data as listed in the top row of the figure. The average values can be found in Tables 3 and 4. The widths of the boxes illustrate the uncertainties in the predicted properties of MLIPs associated with both data selection and random seeds. More precisely, for the periodic MLIPs, these correspond to committee errors obtained from ref 13, while in the case of our cluster MLIPs, they are given as the min/max values from the committees (jointly computed over all cluster data sets). For the cluster-based MLIPs, the large variations in the resulting densities and diffusion coefficients shown in the figure are coupled to the sensitivities of the MLIPs to the specific selection of data points present in the training sets, i.e. differences between the Clusters-Large, Clusters-Medium, Clusters-Small training sets,and between the subsample variants of the latter two; this becomes a crucial matter when cluster data needs to be extrapolated to represent bulk liquid scenarios. Altogether, the figure illustrates that our MLIPs fitted to periodic data can be used to compare functionals, while the cluster-based models produced in this study yield too large uncertainties to make such a comparison meaningful. Experimental values for both EC and EMC have been indicated with dashed lines.

Here, we used only three functionals, but there are, as mentioned, innumerable more DFT functionals to be tested in the search of adequately predictive functionals for studies of diffusion-dependent phenomena of organic liquids. Such systematic tests are beyond the scope of the current study, but our results demonstrate that access to the MACE machinery, and taking training seed variation into account, will make it feasible to perform comprehensive benchmark studies of a large number of DFT functionals, e.g., against experimental observations.

We have shown that our MACE-fitted models trained on periodic data for the four EC:EMC systems are all stable. The resulting densities come out to be quite similar for the three functionals, while the diffusivities depend more strongly on the choice of DFT method, even for the seemingly similar functionals used here (see Figure 5 for an overview). Incidentally, compared with available experimental measurements for pure EC and EMC (Table 3), we note that the three selected DFT methods either reproduce available experimental densities or diffusion coefficients fairly well, but never both. Generating reference data at higher quantum-mechanical levels such as hybrid-DFT or post-HF wave function methods easily becomes prohibitively expensive for periodic systems. This raises an important question: can we train MACE MLIPs on molecular clusters for our EC:EMC systems and predict properties in the condensed phase? EC and EMC data are actually already available at the fourth rung (“meta-GGA and hybrid”) ωB97X-D3 level through the work of Dajnowicz et al.^23^ In the next section, we explore such possibilities using a strategy similar to the one we used here for the periodic systems but using a committee of models.

**Table 3:** Summary of the Densities and Finite Size-Corrected Diffusion Coefficients of the Pure Solvents; Experimental Values Have Been Included as a Perspective; the Corrections Were Determined Using η_EC_ = 1.93 × 10^–3^ Pa s Taken from ref 43 and η_EMC_ = 6.5 × 10^–4^ Pa s Taken from ref 44; the Densities Are Reported with Mean and Standard Error across a Committee as Reported in ref 13, whereas the Diffusivity Is Reported with a 95% Confidence Level in the Linear Fit

solvent	training set	temperature /K	density/g cm^–3^	diff. coeff. (*D*_∞_) /1 × 10^–6^ cm^2^ *s* ^–1^
EC	Periodic/PBE-D3	313	1.23(0.02^a^)	8.53 ± 0.03
	Periodic/PBE-D2	313	1.34(0.02^a^)	6.41 ± 0.01
	Periodic/B97-D3	313	1.30(0.02^a^)	5.61 ± 0.01
	expt.^b^	313	1.312	8.0
				
EMC	Periodic/PBE-D3	298	0.97(0.02^a^)	20.49 ± 0.06
	Periodic/PBE-D2	298	1.05(0.02^a^)	17.70 ± 0.06
	Periodic/B97-D3	298	1.09(0.02^a^)	10.04 ± 0.02
	expt.^c^	298	1.006	18.3

a The error represents the standard error of the mean prediction made using a five-membered committee of models evaluated at 500 K. This value was taken from ref 13.

b The density was computed from a specific gravity of 1.322 at 313 K^45^ and the diffusivity comes from ref 43.

c The density value was taken from the Merck webpage and the diffusivity from personal communication with Prof. Clare P. Gray.

### 3.2 Property Variability from MLIPs Fitted on Cluster Data

Here, a number of MLIP models were trained by us based on the extensive ωB97X-D3 level cluster training set developed by Dajnowicz et al.^23^ intended for organic electrolytes and consisting of organic solvent molecules such as EC, EMC, and a few others (see Figure 2). We essentially follow the procedure used for the periodic MLIPs in Section 3.1 with a couple of exceptions. First a committee of Clusters-Large MLIPs is trained with three different seeds and we monitor the variations in predicted properties resulting from those MLIPs. Then, in order to determine whether the skewness of the data toward EC (Figure 2) has a negative impact, we harmonize the data by partitioning it into smaller subsets and perform a similar analysis using committees of MLIP predictions.

Figure 6 shows committee averages of densities and diffusion coefficients obtained from cluster-based MLIPs for the various EC:EMC compositions, and Table 4 lists the averages (for the pure liquids) and their associated uncertainties. The committee averages were computed jointly over both the various seeds and data composition for each size of the data sets. The variations in predicted properties with respect to either the seed or the data set samples for all compositions are presented in the SI. The MD-simulated liquid densities and diffusion coefficients from the Cluster-data-trained and Periodic-data-trained MLIPs are compared in Figure 5.

**Figure 6:**
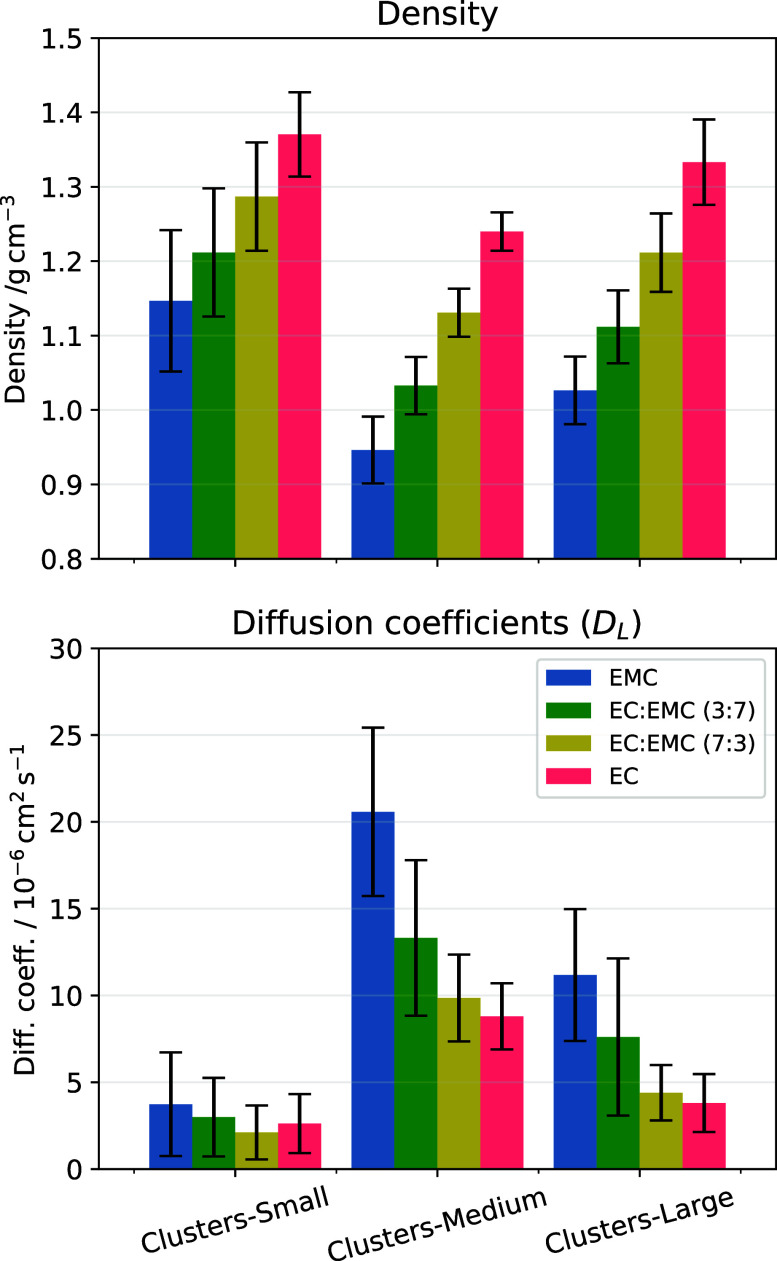
MD simulations results with the Clusters-Small/ωB97X-D3 and Clusters-Medium/ωB97X-D3 and Clusters-Large/ωB97X-D3 MLIPs. The error bars show committee averages calculated jointly over three MLIP seeds (for all three training set sizes) and over three data set samples for the Clusters-Small/ωB97X-D3 and Clusters-Medium/ωB97X-D3 training sets. With the exception of pure EC, which was simulated at 313 K, all other compositions were simulated at 298 K.

**Table 4:** Summary of the Densities and Finite Size-Corrected Diffusion Coefficients of the Pure Solvents; Experimental Values Have B Included as a Perspective; the Corrections Were Determined Using η_EC_ = 1.93 × 10^–3^ Pa s Taken from ref 43 and η_EMC_ = 6.5 × 10^–4^ Pa s Taken from ref 44; the Values are Reported with Mean and Coefficient of Variation (CV in %), Defined as SD/Mean × 100

solvent	training set	temperature /K	density/g cm^–3^		diff. coeff. (*D*_∞_)/1 × 10^–6^ cm^2^ *s* ^–1^	
			mean	CV %	mean	CV %
EC	Clusters-Small/ωB97X-D3	313	1.37	4.1	4.16	41
	Clusters-Medium/ωB97X-D3	313	1.24	2.1	10.27	19
	Clusters-Large/ωB97X-D3	313	1.33	4.3	5.35	31
	expt.^a^	313	1.312		8.0	
						
EMC	Clusters-Small/ωB97X-D3	298	1.15	8.3	8.12	37
	Clusters-Medium/ωB97X-D3	298	0.95	4.8	24.69	20
	Clusters-Large/ωB97X-D3	298	1.03	4.4	15.49	25
	expt.^b^	298	1.006		18.3	

a The density was computed from a specific gravity of 1.322 at 313 K^45^ and the diffusivity comes from ref 43.

b The density was taken from the Merck webpage and the diffusivity from personal communication with Prof. Clare P. Gray.

Before analyzing our results in more detail, we note that all of our MLIPs fitted to cluster data are capable of producing stable MD trajectories (1 ns long). This demonstrates the impressive stability of MACE MLIPs and the modest requirement for data amount, as pointed out in ref 13.

Starting with the Clusters-Large-MLIPs, we immediately observe that there are significant variations in the predicted macroscopic quantities across the committee, with an approximately 4% variation in the density and a 25%–30% variation in the diffusion coefficients. These variations are substantially larger than what was reported by Gong et al.^24^ who attempted to train MLIPs starting from the data of Dajnowicz et al.^23^ In their work, stable MLIPs could only be obtained after three rounds of active learning, which required a considerable amount of data to be added to the Clusters-Full data set. Dajnowicz et al. did not address committee variations, but their values for densities and diffusion coefficients of the pure EC and EMC solvents lie within two standard deviations from the committee average of our Clusters-Large-MLIPs. It should be noted that the MLIPs used in refs 24 and 23 incorporated long-range electrostatics, which may assist in making out-of-domain extrapolation more robust. The importance of long-range information is supported by the recent findings of Gawkowski et al.^19^ where successful out-of-domain transfer could be achieved when a bulk-trained MLIP was fine-tuned on cluster data, i.e., where the MLIP had thus previously seen related structures in a bulk context.

Many of the structures added by Gong et al. consisted of more than one hundred atoms. The largest clusters (in terms of the number of atoms) in our Clusters-Large data set contain 90 atoms, but there are only 16 such instances. In perspective, the number of structures with 90 or more atoms added by Gong et al. were in the tens of thousands. Thus, whether the significantly smaller committee variation obtained by Gong et al. originates from the added electrostatics in their model or from the data added in their active learning process is not clear. The large uncertainties of the committee values from the Cluster-trained MLIPs in Figures 5 and 6 illustrate that even in the case of these seemingly unproblematic organic molecules, cluster data can present a challenging fitting task compared to periodic data if the end purpose is to simulate bulk liquids. RDF-type plots in Figure S2 show that most bulk-like atomic environments in the Clusters-Full data set have coordinations that are much lower than those in the liquid (see the SI for a detailed analysis). The reason for our large committee variations is likely the result of at least one of the two effects illustrated in Figure 7.

**Figure 7:**
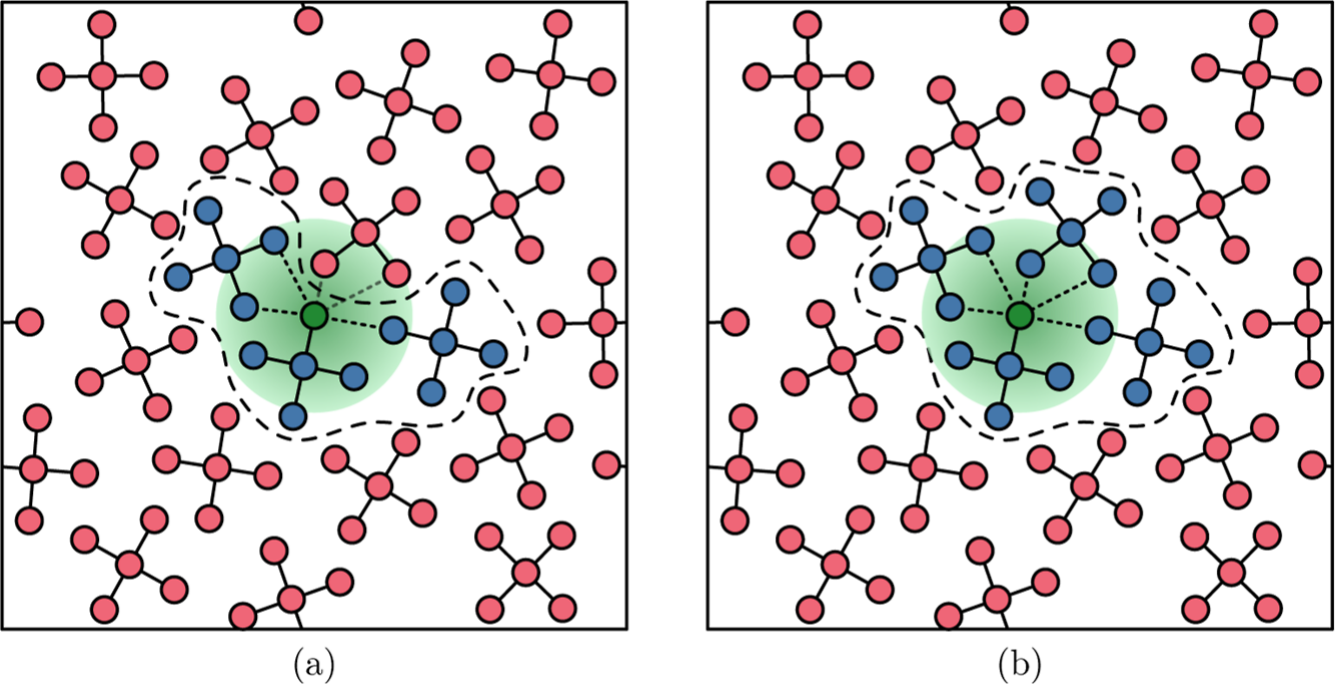
Illustration of two factors that will influence the extrapolation capacity of a cluster-trained MLIP, as a result of the cluster extraction for the training set generation. In Figure (a) a cluster is cut out from the liquid (the dashed line). First, the removal of the red atoms (molecules) is an effective truncation of long-range interactions. Second, the green atom is seen to not even have a full liquid-like atomic coordination within a typical MLIP cutoff (illustrated by the green disc). MLIPs trained on data where a majority of clusters are similar to the one in (a) will likely lack the necessary information to express the full structural fingerprint of an atom embedded in a liquid; this means that out-of-domain extrapolation will come into play when such MLIPs are used in subsequent liquid MD simulations. In contrast, (b) shows a larger cluster where at least some of the atoms have full liquid-like atomic coordination, but still lack long-range interactions.

The figure illustrates the process of selecting clusters from a liquid to serve in a training set for MLIP generation, and the implication of this seection when the MLIP is subsequently applied in periodic MD simulations. Clearly, removing all the red molecules in the two figures will result in the loss of long-range interactions which can provide important contributions to energies and forces (Figure 7). The first effect stems from local coordination around the atoms inside a cluster. If we consider the local atomic environment defined by the green disc in Figure 7a, we know that a liquid will have a characteristic average structure around each atom. If we train an MLIP on data where the intermolecular coordination (within the green disc) is un-bulk-like compared to a liquid the sudden appearance of atoms in these regions during simulation may result in uncertain predictions of energies and forces.

Regarding the representation of structures, we note that the original Clusters-Full data set as well as our Clusters-Large data set contains a disproportionate amount of EC monomers and dimers. The importance of adequate data representation was also highlighted in the work of Goodwin et al.^46^ who trained MLIPs on ionic liquids with dissolved salts of different concentrations and found that training on the extreme concentrations (the smallest and the largest, together) and then predicting on medium concentrations turned out to be far more challenging than doing the reverse.

To mitigate disturbing influences from skewness in the training data, we down-sampled (size reduction) the Clusters-Large data set into successively smaller training sets.

For this reason, we created different variations of these subsamples. We also examined the effect of different training seeds in the same way as for the Clusters-Large MLIPs. As summarized in Section 2.1, the down-sampling of the Clusters-Large data set was performed in two steps where, in each step, 25% of the previous (larger) data set was selected with the aim of making it more bulk-like and more compositionally balanced. This resulted in two additional data set sizes: Clusters-Medium which contained 25% of Clusters-Large, and Clusters-Small which contained 25% of Clusters-Medium. Figure 2 shows the different molecular compositions of the various data set sizes. The transition from Clusters-Large to Clusters-Medium is associated with a considerable reduction in pure EC clusters. The strategy behind generating the Clusters-Medium composition was to include a more diverse set of atomic environments than only EC and EMC could provide. Despite not being part of the liquid simulations, the other molecules (see caption of Figure 2) are similar to either EC or EMC. Therefore, from the atomic-centered perspective of the MACE-MLIPs, many of the atomic environments present in these clusters are likely relevant also for EC and EMC. For example, clusters containing DEC may provide examples of atomic environments that are relevant also for EMC—which happens to be poorly represented in the Clusters-Full and Clusters-Large data sets. The motivation behind the reduction from Clusters-Medium to Clusters-Small was based on similar grounds. Three copies were then made of each reduced data set which, for each data set size, all shared the same relative molecular composition but not exactly the same selection of clusters. To be precise, three copies were made of the Clusters-Medium data set which all shared the composition depicted in the second panel of Figure 2, but which did not share exactly the same cluster identities throughout. The same procedure was carried out for the Clusters-Small data set, resulting in a total of seven data sets (3 × Clusters-Small + 3 × Clusters-Medium + 1 × Clusters-Large).

Figures 5 and 6 display similar large uncertainties for properties produced by the MLIPs trained on the smaller subsets of the cluster data, as found for the Cluster-Large MLIPs discussed above. The error bars are either touching or overlapping, making the results from the different MLIPs trained on different data set sizes statistically indistinguishable from one another. For completeness, MLIPs were also trained on the three variants of the Clusters-Small and of Clusters-Medium data sets that had been labeled with the B97-D3 functional. As the predictive quality of these MLIPs (see Figures S6 and S7 in the SI) did not improve, we conclude that the uncertainties of the cluster-based data is not coupled to the level of theory used to label the structures. A direct comparison among the Periodic/B97-D3, Clusters-Small/B97-D3, and Clusters-Medium/B97-D3 models, shown in Figure S8, highlights this.

In conclusion, the differences in predicted macroscopic quantities in our cluster fitted models are much larger than those from models fitted on different functionals for periodic data. These cluster data sets can therefore not be used to directly extend our functional comparison without further augmentation/modification of the data. This has important implications to both tailored MLIPs and foundational models. Whether or not explicit long-range interactions can serve to counter the variations that are due to the training seed remains to be seen and may be the subject of a future study.

## 4 Concluding Remarks

The overall context of our study is the benchmarking of the DFT functionals. We have presented a strategy to enable the comparison of DFT functionals with respect to their ability to describe macroscopic liquid properties such as density and diffusivity here for EC:EMC mixtures. By training MACE-MLIPs on periodic DFT-GGA data from the literature and subsequently relabeling the data using different GGA functionals, we were able to meaningfully compare both the densities and diffusion coefficients from long (and stable) MD simulations between functionals without having to resort to active learning. Here, only three functionals were compared as a proof-of-concept for the approach, but the same strategy can, in principle, be adopted for any functional applicable to periodic systems.

The second part of our study deals with MLIPs trained on cluster data, starting from data available in the literature, namely, a large cluster data set of organic molecules labeled at the ωB97X-D3 level of theory. Given the current interest in the fitting of MLIPs to cluster data, we highlight aspects that need to be taken into account when cluster-based MLIPs are trained for use in bulk simulations. We demonstrate that our MLIPs trained on clusters exhibit large uncertainties in densities and diffusivities. We attribute this problem to the lack of representative bulk coordination in the training data and/or long-range interactions, a challenge which translates into an out-of-domain extrapolation problem when the cluster-based models are being applied to MD bulk simulations (cf. Figure 7). Our study should be of particular relevance within the context of transfer learning on high-level QM data, a field that is quickly gaining ground in the MLIP community.

While the resulting cluster-based MACE-MLIPs produced stable MD trajectories, the resulting variations of densities and diffusivities across model committees were significantly larger than with the periodic-data-based MLIPs—in fact, too large to make meaningful comparisons between functionals. Because of the impressive capability of MACE to produce stable MLIPs even for small/unbalanced training sets, treating MACE-MLIP stability as a quality indicator may be treacherous.

## Supplementary Material



## Data Availability

The training data was published in our previous work^8^ and by Dajnowicz et al.^23^ The MD trajectories generated here are freely available and accessible through the github-page: https://github.com/viktorsvahn/Limits_of_cluster_MLIPs, along with Jupyter-notebooks that contain analysis scripts.

## Notes

Details about MLIP performance measured in terms of structure, density, and diffusion coefficients; and detailed analysis about the compactness of clusters in the cluster-based training sets (PDF)
