# Supplementary material for: Limitations of Cluster-Trained MLIPs for Liquid Density and Diffusivity
Source: J Chem Theory Comput. 2026 Mar 31;22(7):3660–71. doi: 10.1021/acs.jctc.5c02043 (PMC13085234; doi:10.1021/acs.jctc.5c02043)
Supplement: Supplementary file 1 [file ct5c02043_si_001.pdf]

Supporting information for:
Limitations of cluster-trained MLIPs for liquid density and diffusivity

Viktor Svahn[1], Ioan-Bogdan Magdău[2], Samuel P. Niblett[3,4], Gábor Csányi[5], Kersti Hermansson[1], Jolla Kullgren[1]

March 24, 2026

[1]Department of Chemistry-Ångström, Uppsala University, Box 538, S-75231 Uppsala, Sweden.
[2]School of Natural and Environmental Sciences, Newcastle University, Newcastle upon Tyne, NE1 7RU, UK.
[3]Yusuf Hamied Department of Chemistry, University of Cambridge, Lensfield Road, Cambridge, CB2 1EW, UK.
[4]Dassault Systémes BIOVIA, 334 Cambridge Science Park, Cambridge CB4 0WN, UK.
[5]Engineering Laboratory, University of Cambridge, Cambridge, CB2 1PZ UK.

S1 MLIPs

This section includes training errors for the various MLIPs trained in this work. Table S1 shows energies (per atom) and forces evaluated over the training and validation sets. Figure S1 shows correlation of forces that have been integrated over a small increment. The correlation between the training labels and the the predictions are effectively the same as a regular force correlation, but the resulting RMSE has units of energy instead.

Table S1: Training (Tr.) and validation (Val.) RMSE's in energy per atom and forces of all MLIPs used in this study. The training sets are shown as `Dataset-`n`/Functional`, where n denotes the numbering of the dataset subsample.

		RMSE Energy per atom /meV		RMSE Force /meV Å^{-1}	
Training set	Seed ID	Tr.	Val.	Tr.	Val.
`Periodic/PBE-D3`	1	0.9	0.7	10.8	18.5
`Periodic/PBE-D2`	1	1.1	0.7	11.2	17.7
`Periodic/B97-D3`	1	2.7	1.0	10.6	17.5
`Clusters-Small-1/`ω`B97X-D3`	1	0.4	0.4	7.9	21.6
`Clusters-Small-1/`ω`B97X-D3`	2	0.4	0.5	8.9	18.4
`Clusters-Small-1/`ω`B97X-D3`	3	0.4	0.5	7.9	19.2
`Clusters-Small-2/`ω`B97X-D3`	1	0.4	0.4	7.8	16.7
`Clusters-Small-3/`ω`B97X-D3`	1	0.4	0.6	8.6	20.7
`Clusters-Small-1/B97-D3`	1	0.3	0.5	8.0	21.8
`Clusters-Small-2/B97-D3`	1	0.4	0.5	8.5	17.5
`Clusters-Small-3/B97-D3`	1	0.4	0.4	9.0	17.9
`Clusters-Medium-1/`ω`B97X-D3`	1	0.5	0.4	7.7	13.5
`Clusters-Medium-1/`ω`B97X-D3`	2	0.3	0.3	7.0	12.0
`Clusters-Medium-1/`ω`B97X-D3`	3	0.3	0.3	7.1	11.3
`Clusters-Medium-2/`ω`B97X-D3`	1	0.3	0.3	7.0	12.0
`Clusters-Medium-3/`ω`B97X-D3`	1	0.3	0.3	7.1	11.3
`Clusters-Medium-1/B97-D3`	1	0.3	0.4	8.3	13.6
`Clusters-Medium-2/B97-D3`	1	4.0	0.3	7.1	10.6
`Clusters-Medium-3/B97-D3`	1	4.0	0.3	7.1	11.0
`Clusters-Large/`ω`B97X-D3`	1	0.4	0.4	8.9	11.4
`Clusters-Large/`ω`B97X-D3`	2	0.4	0.4	8.4	10.5
`Clusters-Large/`ω`B97X-D3`	3	0.4	0.4	8.4	9.9
Total number of models:	22				

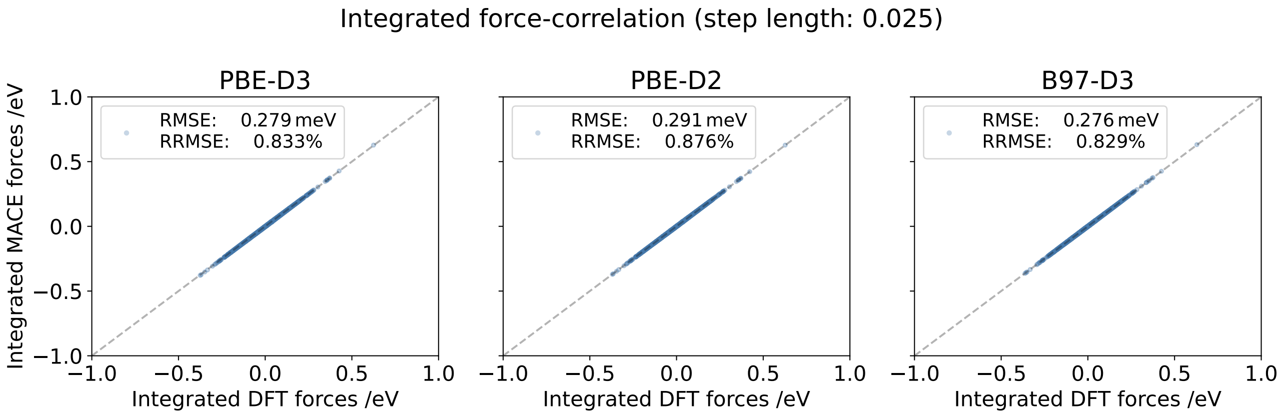

Figure S1: Correlation between integrated forces from prediction (y-axis) and the labels used in the training (x-axis). The forces were integrated over a constant step of 0.025 24 Å, which is the average (over ten time steps) step length per time step per atom in subsequent NPT simulations (Fig. S3).

S2 Structural comparison between periodic- and cluster-based training sets

The radial distribution function (RDF) describes the average pair-wise distance between neighbouring particles at a given distance, and can be defined as [1]

$$g(r) = \frac{\langle n(k) \rangle}{4\pi r^2 \, \mathrm{d}r \, \rho} \tag{S1}$$

where ρ is the number density of the bulk, $n(k)$ is the average number of neighbours at distances in the kth bin which spans between $r - \frac{1}{2}\delta r$ and $r + \frac{1}{2}\delta r$ and where the average $\langle \cdot \rangle$ is taken over a whole trajectory (or dataset). The presence of the constant ρ in equation (S1) means g can only be evaluated inside a well-defined geometry, making equation (S1) impractical for non-periodic structures. However, $\langle n(k) \rangle$ is well-defined also for non-periodic systems. We can therefore define a RDF analog for our cluster data by using $n(k)$ and a reference density, ρ_ℓ. However, in order to make fair comparison of the local structure between the `Clusters-Large` dataset and liquids, we also attempt to assess existence—rather than prevalence—of denser pair-coordinations. Whether or not the average $\langle n(r) \rangle$ is taken over one bin at a time, or across all bins at the same time is a matter of perspective. By adopting the former we may define

$$g'(r) = \frac{\max_k n(k)}{4\pi r^2 \, \mathrm{d}r \, \rho_\ell} \tag{S2}$$

which will generate an RDF of the *greatest* pair-coordination in every bin, k, across the whole dataset. Here ρ_ℓ puts the distribution in relation to a liquid reference. Therefore, by comparing g to g', we can see if the greatest density of every bin across a non-periodic dataset is comparable to that of a liquid (wherever $g' < g$ the non-periodic dataset fails to match the density of a liquid).

The left panel of Fig. S2 shows the average inter-molecular neighbour density $\langle n(r) \rangle$, where r denotes the centre of a bin, k. The `Clusters-Large` dataset and the liquid are clearly not similar when it comes to inter-molecular coordination because the number of dimers far outweigh the number of clusters with more molecules. The right panel compares RDFs between liquid and `Clusters-Large` showing that, on average, the difference between the clusters and the liquid is considerable and that the highest densities found within `Clusters-Large` near the model cut-off are nowhere near the corresponding density of the liquid (gray area).

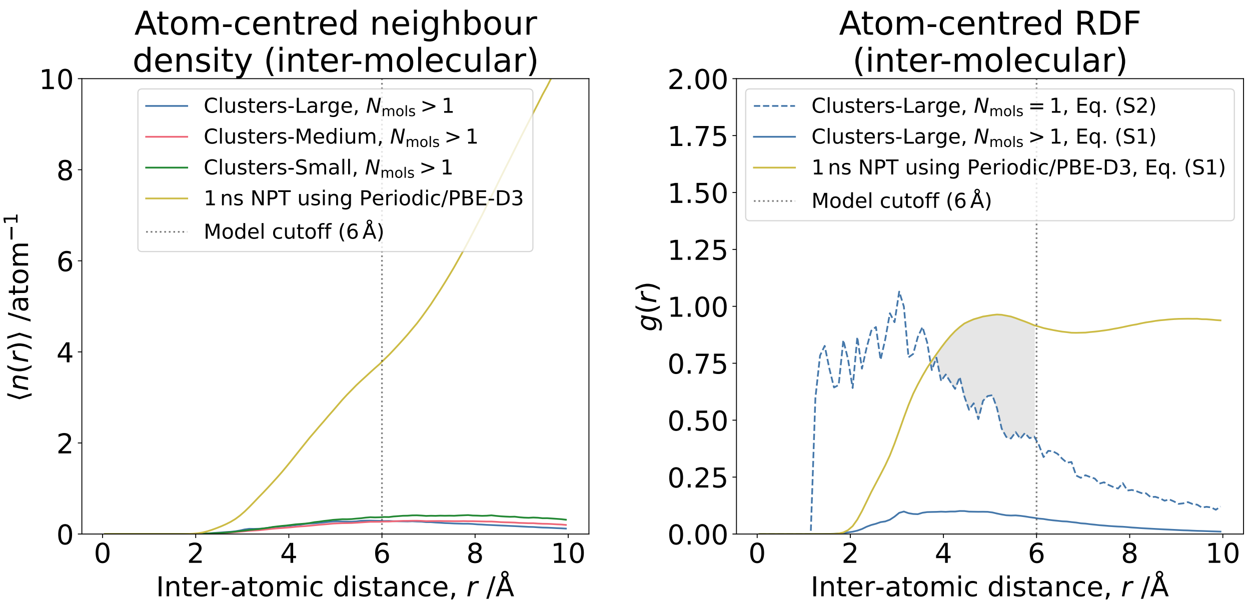

Figure S2: Structural comparisons between liquid (yellow) and clusters. The left panel shows the average neighbour density around an atom, taking only inter-molecular contributions into account. The right panel compares $g(r)$ between a cluster-based dataset and a liquid reference using Eqs (S1) and (S2), respectively. The vertical dotted lines indicate the MLIP cut-off used during the training.

S3 Thermodynamic properties of liquids

This section contains all the results obtained from the cluster models with visuals followed by tabulated data. The visuals includes the densities and uncorrected diffusion coefficients. The tables include some uncertainties in the form of standard deviations for the densities and the error in the slope fit of the diffusion coefficients estimated with a two-sided t-test and the R^2-value of said fit. Furthermore, since the diffusion coefficient was fitted on all the data points in the linear region of the log-log plots, the starting time for this region along was included. For details regarding this approach, see the methods section. To highlight the differences between the MLIPs in the fitting results, the R^2-values have been coloured. The limits for this colouring are arbitrary and should be considered as guides for the eye.

S3.1 Properties resulting from periodic data

This section contains all results associated with the periodic data sets are shown in Fig. S3. Due to the close relationship with a previous paper, results that were obtained using the Gaussian approximation potential (GAP) have also been included in this figure. The numbers associated with Fig. S3 have been included in Table S3.

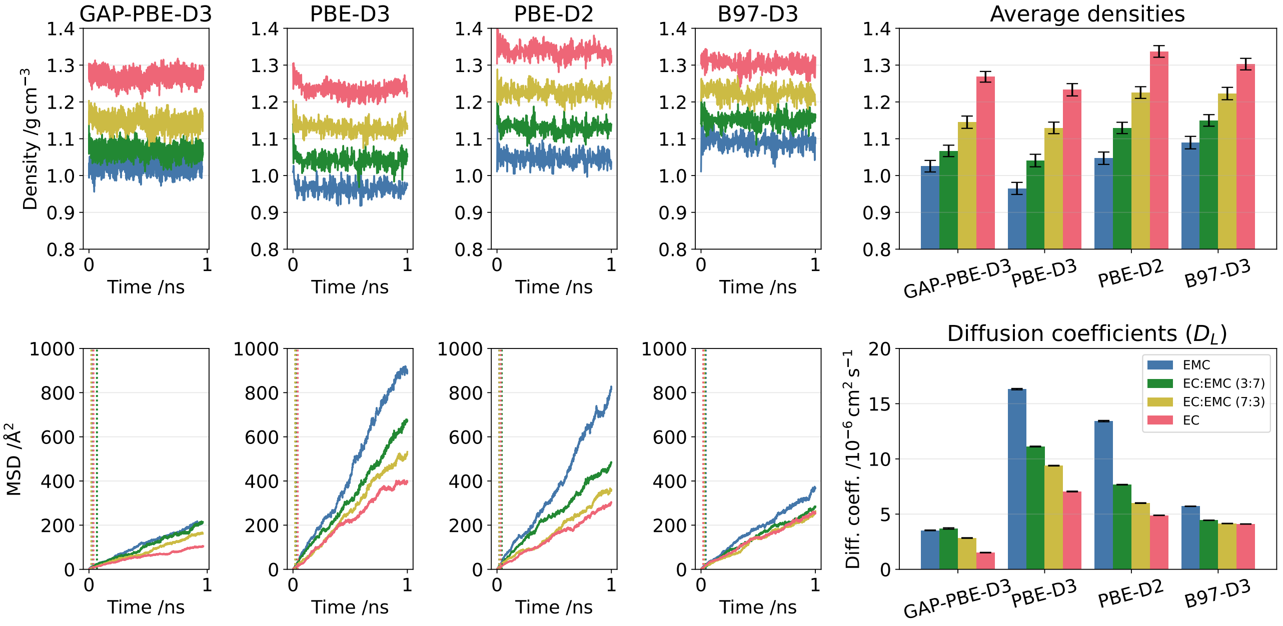

Figure S3: Effects on the MD-generated properties of changing the DFT functional. Results from MD simulations with three different MLIPs are shown, all trained on the `Periodic` data set structures but labelled with PBE-D3, PBE-D2 and B97-D3 values, respectively. The colour scheme refers to the compositions in the MD simulations and is explained in the legend of the upper left frame. The first three columns display the time evolution of the system densities (top) and of the mean-squared-displacements (MSD) (bottom). The bar diagrams in the rightmost column display the corresponding average density (top frame) and the diffusion coefficients (bottom frame) fitted from the MSD curves, starting from the respective vertical lines in the MSD time evolution graphs. Here the GAP results were taken from Ref. [2].

Table S2: Summary of MD results obtained from the `Periodic`-data set labelled with different functionals. With respect to the R^2-values, the colour is red when below 0.8; orange when below 0.9; and yellow when below 0.95.

Composition	Temperature /K	Density /g cm^{-3}	Diff. coeff. (D_L) /1 × 10^{-6} cm^2 s^{-1}	Diff. slope fit R^2	Diffusion start time /ns	Diffusion start time R^2	
`Periodic/PBE-D3`	EMC	298	0.97 ± 0.16	16.33 ± 0.60	0.9906	0.025	0.9647
	EC:EMC (3:7)	298	1.04 ± 0.17	11.13 ± 0.32	0.9942	0.030	0.9785
	EC:EMC (7:3)	298	1.13 ± 0.16	9.39 ± 0.28	0.9936	0.022	0.9484
	EC	313	1.23 ± 0.17	7.02 ± 0.31	0.9865	0.058	0.9631
`Periodic/PBE-D2`	EMC	298	1.05 ± 0.17	13.43 ± 0.59	0.9865	0.021	0.9725
	EC:EMC (3:7)	298	1.13 ± 0.15	7.66 ± 0.25	0.9929	0.043	0.9186
	EC:EMC (7:3)	298	1.23 ± 0.16	6.00 ± 0.29	0.9839	0.020	0.9393
	EC	313	1.34 ± 0.16	4.89 ± 0.14	0.9941	0.034	0.9542
`Periodic/B97-D3`	EMC	298	1.09 ± 0.17	5.71 ± 0.17	0.9939	0.041	0.9768
	EC:EMC (3:7)	298	1.15 ± 0.16	4.44 ± 0.17	0.9904	0.039	0.9189
	EC:EMC (7:3)	298	1.22 ± 0.17	4.15 ± 0.17	0.9883	0.030	0.9764
	EC	313	1.30 ± 0.16	4.10 ± 0.13	0.9927	0.020	0.9631

S3.2 Properties resulting from cluster data

This section contains all results associated with the cluster based training sets. Figs S4 and S5 shows the densities and the diffusivities, respectively, of the different MLIPs trained on cluster-data at the ωB97X-D3 level of theory. The associated numbers can be found in Tables S3-S5.

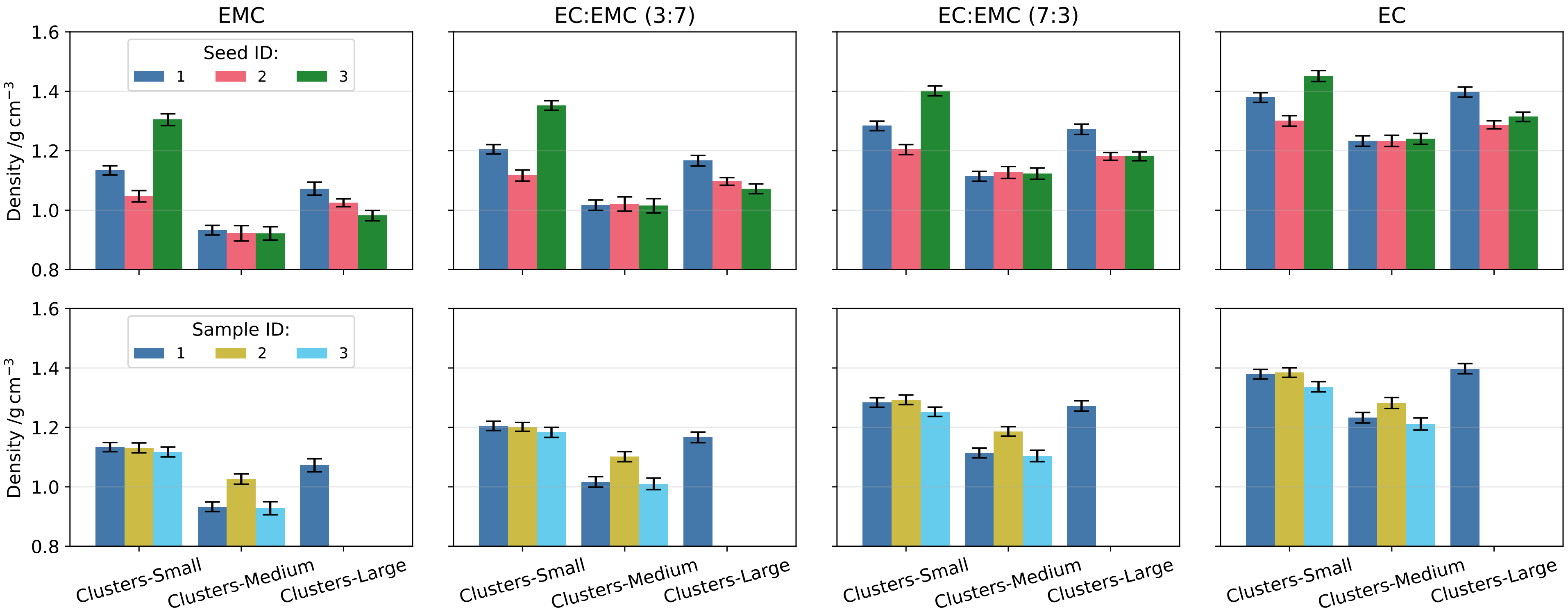

Figure S4: Densities of various compositions of EC/EMC solvent after a 1 ns NPT simulation using the `Clusters-Small`, `Clusters-Medium` and `Clusters-Large` models (labelled with ω`B97X-D3`). The top row shows variations with respect to the random seed and the bottom with respect to different data set samples.

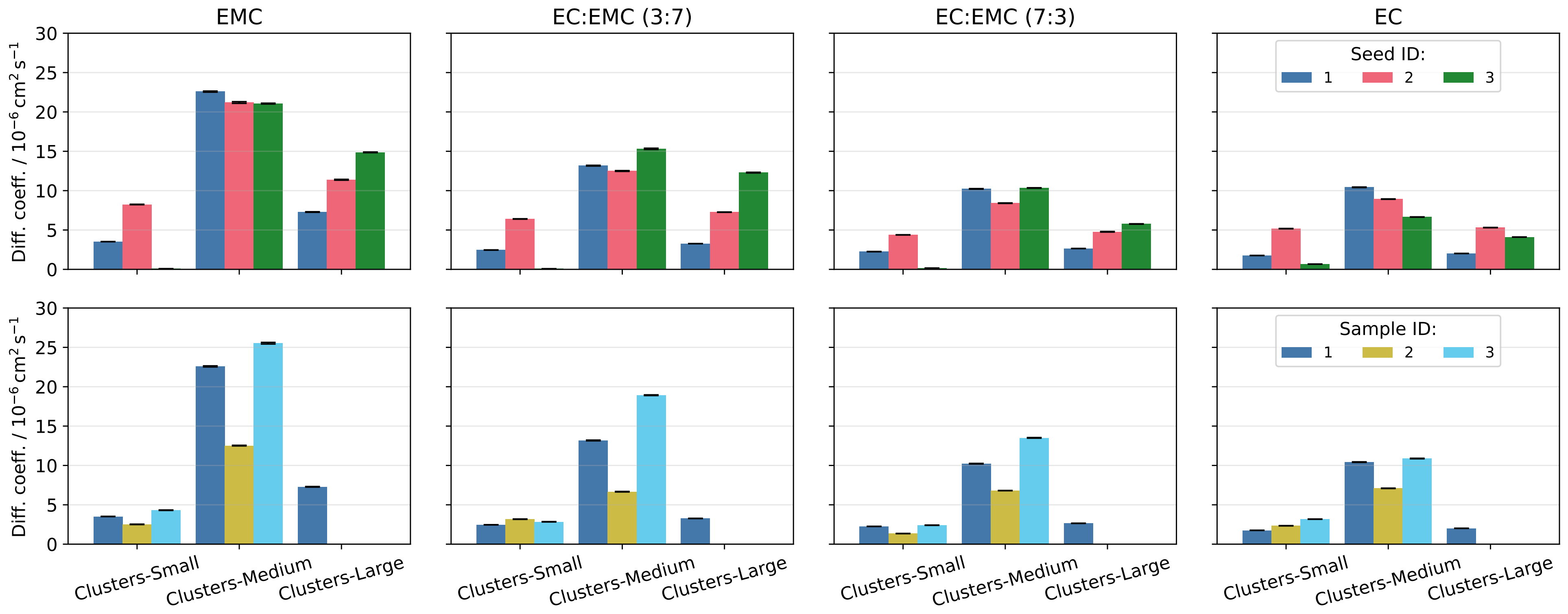

Figure S5: Diffusion coefficients of various compositions of EC/EMC solvent after a 1 ns NPT simulation using the `Clusters-Small`, `Clusters-Medium` and `Clusters-Large` models (labelled with ω`B97X-D3`). The top row shows variations with respect to the random seed and the bottom with respect to different data set samples. These coefficients have not been corrected for finite size effects.

Table S3: Summary of MD results obtained from `Clusters-Small`/ω`B97X-D3`. With respect to the R^2-values, the colour is red when below 0.8; orange when below 0.9; and yellow when below 0.95.

Seed ID	Sample ID	Composition	Temperature /K	Density /g cm^{-3}	Diff. coeff. (D_L) /1×10^{-6} cm^2 s^{-1}	Diff. slope fit R^2	Diffusion start time /ns	Diffusion start time R^2
1	1	EMC	298	1.13 ± 0.16	3.52 ± 0.12	0.9925	0.109	0.8189
1	1	EC:EMC (3:7)	298	1.21 ± 0.16	2.46 ± 0.12	0.9839	0.096	0.8885
1	1	EC:EMC (7:3)	298	1.28 ± 0.16	2.26 ± 0.13	0.9769	0.058	0.9094
1	1	EC	313	1.38 ± 0.16	1.76 ± 0.09	0.9826	0.114	0.9154
1	2	EMC	298	1.13 ± 0.17	2.51 ± 0.25	0.9532	0.130	0.8938
1	2	EC:EMC (3:7)	298	1.20 ± 0.15	3.17 ± 0.20	0.9783	0.044	0.9213
1	2	EC:EMC (7:3)	298	1.29 ± 0.16	1.35 ± 0.09	0.9767	0.110	0.8472
1	2	EC	313	1.38 ± 0.16	2.34 ± 0.12	0.9849	0.039	0.9646
1	3	EMC	298	1.12 ± 0.17	4.31 ± 0.27	0.9767	0.152	0.8792
1	3	EC:EMC (3:7)	298	1.18 ± 0.17	2.84 ± 0.18	0.9717	0.025	0.9551
1	3	EC:EMC (7:3)	298	1.25 ± 0.16	2.41 ± 0.18	0.9662	0.133	0.9043
1	3	EC	313	1.34 ± 0.17	3.17 ± 0.13	0.9902	0.148	0.8208
2	1	EMC	298	1.05 ± 0.19	8.24 ± 0.26	0.9931	0.043	0.9329
2	1	EC:EMC (3:7)	298	1.12 ± 0.19	6.40 ± 0.21	0.9921	0.024	0.9612
2	1	EC:EMC (7:3)	298	1.20 ± 0.17	4.38 ± 0.12	0.9950	0.087	0.9175
2	1	EC	313	1.30 ± 0.18	5.17 ± 0.20	0.9900	0.096	0.9314
3	1	EMC	298	1.30 ± 0.20	0.08 ± 0.03	0.4649	0.172	0.7082
3	1	EC:EMC (3:7)	298	1.35 ± 0.16	0.08 ± 0.02	0.7645	0.094	0.8153
3	1	EC:EMC (7:3)	298	1.40 ± 0.17	0.16 ± 0.02	0.8930	0.111	0.8237
3	1	EC	313	1.45 ± 0.18	0.66 ± 0.04	0.9783	0.104	0.8263

Table S4: Summary of MD results obtained from `Clusters-Medium`/ω`B97X-D3`. With respect to the R^2-values, the colour is red when below 0.8; orange when below 0.9; and yellow when below 0.95.

Seed ID	Sample ID	Composition	Temperature /K	Density /g cm^{-3}	Diff. coeff. (D_L) /1×10^{-6} cm^2 s^{-1}	Diff. slope fit R^2	Diffusion start time /ns	Diffusion start time R^2
1	1	EMC	298	0.93 ± 0.16	22.58 ± 0.67	0.9939	0.034	0.9408
1	1	EC:EMC (3:7)	298	1.02 ± 0.17	13.17 ± 0.44	0.9923	0.056	0.9523
1	1	EC:EMC (7:3)	298	1.11 ± 0.17	10.22 ± 0.33	0.9932	0.074	0.9583
1	1	EC	313	1.23 ± 0.18	10.42 ± 0.33	0.9931	0.042	0.9404
1	2	EMC	298	1.03 ± 0.17	12.52 ± 0.37	0.9939	0.057	0.9679
1	2	EC:EMC (3:7)	298	1.10 ± 0.17	6.66 ± 0.29	0.9863	0.020	0.9840
1	2	EC:EMC (7:3)	298	1.19 ± 0.16	6.80 ± 0.16	0.9961	0.039	0.9552
1	2	EC	313	1.28 ± 0.18	7.09 ± 0.22	0.9936	0.040	0.9144
1	3	EMC	298	0.93 ± 0.22	25.53 ± 1.00	0.9911	0.192	0.9620
1	3	EC:EMC (3:7)	298	1.01 ± 0.19	18.92 ± 0.43	0.9964	0.063	0.9745
1	3	EC:EMC (7:3)	298	1.10 ± 0.19	13.50 ± 0.37	0.9948	0.036	0.9663
1	3	EC	313	1.21 ± 0.20	10.88 ± 0.31	0.9943	0.034	0.9276
2	1	EMC	298	0.92 ± 0.26	21.20 ± 1.04	0.9832	0.026	0.9697
2	1	EC:EMC (3:7)	298	1.02 ± 0.24	12.50 ± 0.49	0.9901	0.089	0.9411
2	1	EC:EMC (7:3)	298	1.13 ± 0.20	8.41 ± 0.29	0.9916	0.036	0.9491
2	1	EC	313	1.23 ± 0.19	8.92 ± 0.25	0.9946	0.031	0.9775
3	1	EMC	298	0.92 ± 0.22	21.06 ± 0.50	0.9959	0.022	0.9810
3	1	EC:EMC (3:7)	298	1.01 ± 0.24	15.31 ± 0.74	0.9835	0.021	0.9848
3	1	EC:EMC (7:3)	298	1.12 ± 0.19	10.34 ± 0.20	0.9974	0.035	0.9781
3	1	EC	313	1.24 ± 0.18	6.65 ± 0.16	0.9958	0.060	0.9603

Table S5: Summary of MD results obtained from `Clusters-Large`/ω`B97X-D3`. With respect to the R^2-values, the colour is red when below 0.8; orange when below 0.9; and yellow when below 0.95.

Seed ID	Sample ID	Composition	Temperature /K	Density /g cm^{-3}	Diff. coeff. (D_L) /1×10^{-6} cm^2 s^{-1}	Diff. slope fit R^2	Diffusion start time /ns	Diffusion start time R^2
1	1	EMC	298	1.07 ± 0.22	7.29 ± 0.34	0.9849	0.029	0.9759
1	1	EC:EMC (3:7)	298	1.17 ± 0.18	3.27 ± 0.11	0.9923	0.075	0.9437
1	1	EC:EMC (7:3)	298	1.27 ± 0.17	2.65 ± 0.11	0.9872	0.043	0.9738
1	1	EC	313	1.40 ± 0.17	2.02 ± 0.10	0.9857	0.175	0.7127
2	1	EMC	298	1.03 ± 0.13	11.38 ± 0.53	0.9849	0.022	0.9583
2	1	EC:EMC (3:7)	298	1.10 ± 0.13	7.27 ± 0.22	0.9937	0.025	0.9607
2	1	EC:EMC (7:3)	298	1.18 ± 0.13	4.77 ± 0.33	0.9693	0.075	0.9607
2	1	EC	313	1.29 ± 0.13	5.31 ± 0.13	0.9961	0.102	0.9085
3	1	EMC	298	0.98 ± 0.17	14.86 ± 0.39	0.9953	0.044	0.9825
3	1	EC:EMC (3:7)	298	1.07 ± 0.16	12.29 ± 0.42	0.9919	0.020	0.9708
3	1	EC:EMC (7:3)	298	1.18 ± 0.15	5.77 ± 0.23	0.9895	0.074	0.9077
3	1	EC	313	1.31 ± 0.16	4.10 ± 0.15	0.9914	0.057	0.9200

S3.2.1 Properties resulting from a change in functional

This part is concerned with the re-computation of the `Clusters-Small` and `Clusters-Medium` data sets to B97-D3 labels. The summarized data from these particular MLIPs are found in the tables below, whereas the ωB97X-D3 data shown in the plots below can be found in the tables in previous sections. Figs S6 and S7 shows the densities and the diffusivities, respectively, of the different MLIPs trained on cluster-data at the B97-D3 level of theory. The associated numbers can be found in Tables S6-S7.

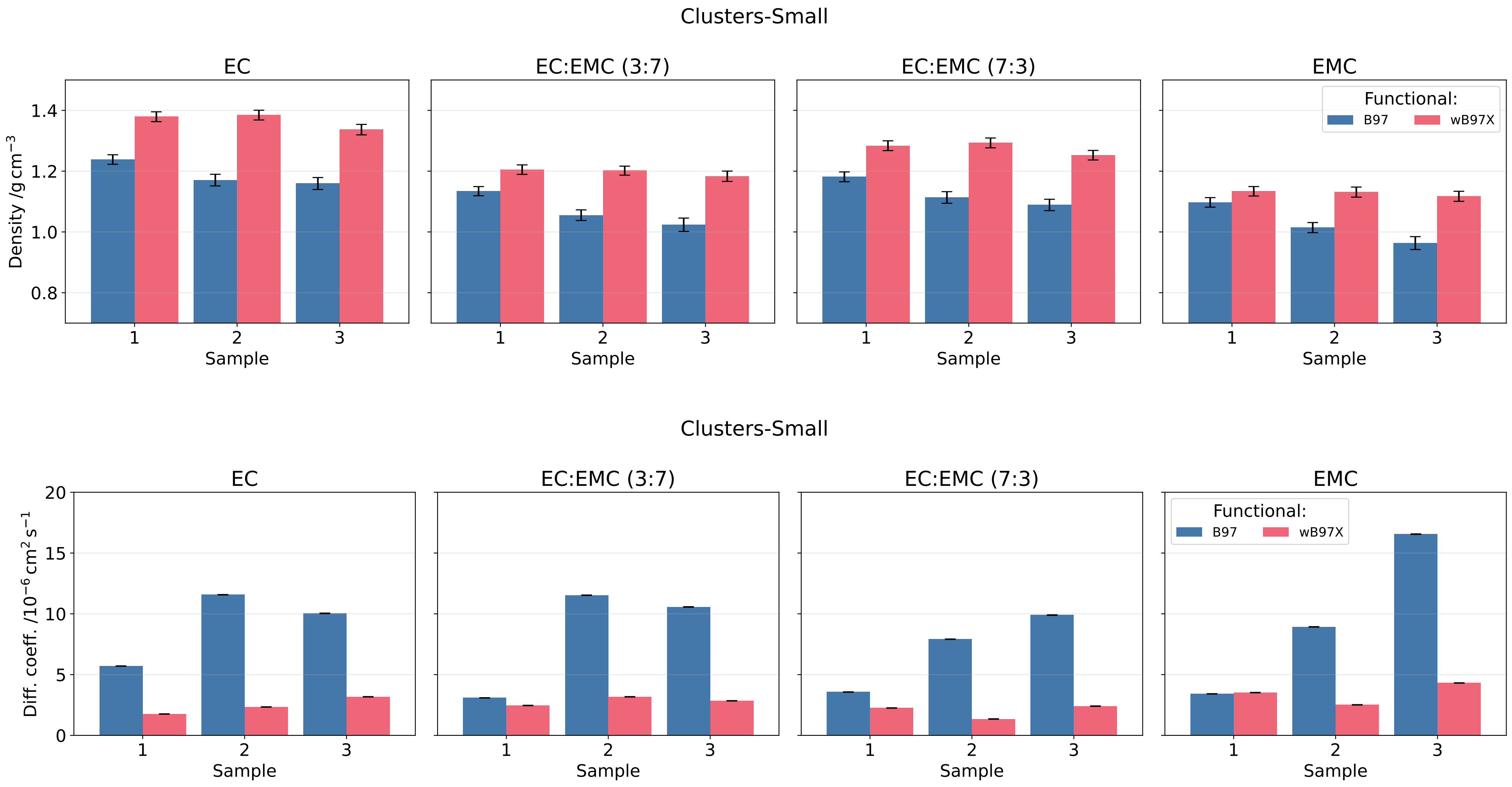

Figure S6: Differences in densities (top) and diffusion coefficients (bottom) between the three different variations of the `Clusters-Small`/`B97-D3` and `Clusters-Small`/ω`B97X-D3` models.

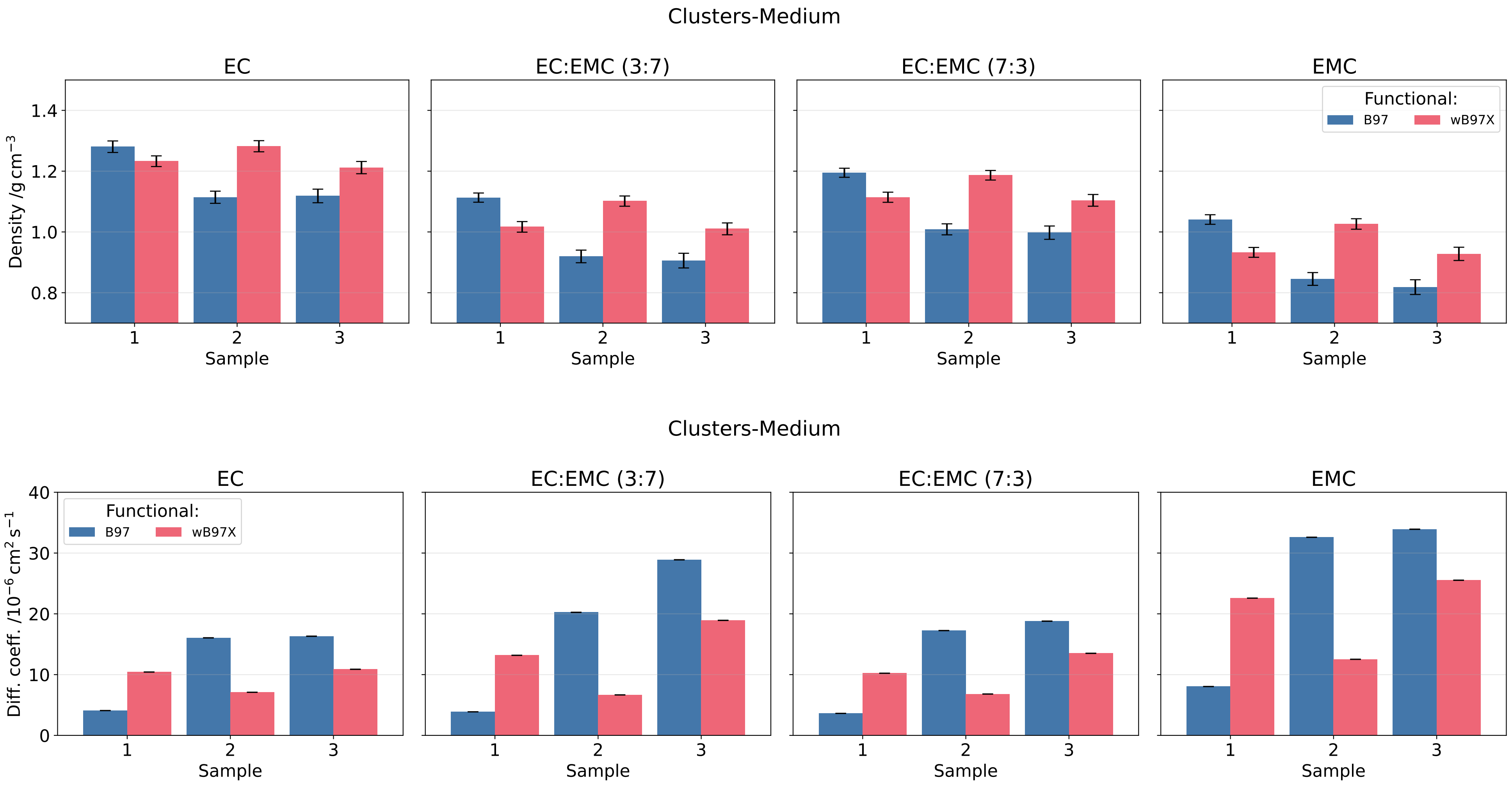

Figure S7: Differences in densities (top) and diffusion coefficients (bottom) between the three different variations of the `Clusters-Medium`/B97-D3 and `Clusters-Medium`/ωB97X-D3 models.

Table S6: Summary of MD results obtained from `Clusters-Small/B97-D3`. With respect to the R^2-values, the colour is red when below 0.8; orange when below 0.9; and yellow when below 0.95.

Seed ID	Sample ID	Composition	Temperature /K	Density /g cm^{-3}	Diff. coeff. (D_L) /1×10^{-6} cm^2 s^{-1}	Diff. slope fit R^2	Diffusion start time /ns	Diffusion start time R^2
1	1	EMC	298	1.10 ± 0.16	3.44 ± 0.13	0.9898	0.029	0.9243
1	1	EC:EMC (3:7)	298	1.13 ± 0.15	3.18 ± 0.19	0.9752	0.066	0.9471
1	1	EC:EMC (7:3)	298	1.18 ± 0.16	3.61 ± 0.12	0.9919	0.031	0.9798
1	1	EC	313	1.24 ± 0.16	5.75 ± 0.20	0.9922	0.065	0.9366
1	2	EMC	298	1.01 ± 0.17	8.94 ± 0.18	0.9972	0.044	0.9458
1	2	EC:EMC (3:7)	298	1.06 ± 0.18	11.35 ± 0.29	0.9957	0.085	0.9225
1	2	EC:EMC (7:3)	298	1.11 ± 0.19	7.89 ± 0.17	0.9966	0.020	0.9873
1	2	EC	313	1.17 ± 0.19	11.44 ± 0.50	0.9869	0.048	0.9656
1	3	EMC	298	0.96 ± 0.21	16.34 ± 0.50	0.9934	0.041	0.9724
1	3	EC:EMC (3:7)	298	1.02 ± 0.22	10.55 ± 0.34	0.9924	0.020	0.9668
1	3	EC:EMC (7:3)	298	1.09 ± 0.19	9.99 ± 0.30	0.9938	0.023	0.9716
1	3	EC	313	1.16 ± 0.20	10.21 ± 0.54	0.9816	0.086	0.9063

Table S7: Summary of MD results obtained from `Clusters-Medium/B97-D3`. With respect to the R^2-values, the colour is red when below 0.8; orange when below 0.9; and yellow when below 0.95.

Seed ID	Sample ID	Composition	Temperature /K	Density /g cm^{-3}	Diff. coeff. (D_L) /1×10^{-6} cm^2 s^{-1}	Diff. slope fit R^2	Diffusion start time /ns	Diffusion start time R^2
1	1	EMC	298	1.04 ± 0.16	7.94 ± 0.26	0.9921	0.024	0.9419
1	1	EC:EMC (3:7)	298	1.11 ± 0.15	3.91 ± 0.14	0.9919	0.069	0.8842
1	1	EC:EMC (7:3)	298	1.19 ± 0.15	3.69 ± 0.15	0.9892	0.039	0.8965
1	1	EC	313	1.28 ± 0.19	4.10 ± 0.23	0.9814	0.163	0.8207
1	2	EMC	298	0.85 ± 0.21	32.49 ± 1.17	0.9910	0.042	0.9910
1	2	EC:EMC (3:7)	298	0.92 ± 0.21	20.57 ± 0.70	0.9920	0.032	0.9470
1	2	EC:EMC (7:3)	298	1.01 ± 0.18	17.32 ± 0.37	0.9967	0.027	0.9714
1	2	EC	313	1.11 ± 0.20	16.01 ± 0.73	0.9865	0.092	0.9717
1	3	EMC	298	0.82 ± 0.24	33.99 ± 0.88	0.9957	0.120	0.9489
1	3	EC:EMC (3:7)	298	0.91 ± 0.24	28.65 ± 1.02	0.9911	0.026	0.9803
1	3	EC:EMC (7:3)	298	1.00 ± 0.22	18.95 ± 0.69	0.9907	0.025	0.9686
1	3	EC	313	1.12 ± 0.22	16.20 ± 0.54	0.9923	0.032	0.9676

S3.3 Comparison between periodic and cluster data

Comparison between MLIPs trained on periodic- and cluster-based data labelled at the B97-D3 level of theory. Given the aspects discussed in the paper, direct comparison between these MLIPs is not justified, and the purpose of Fig. S8 is to highlight this.

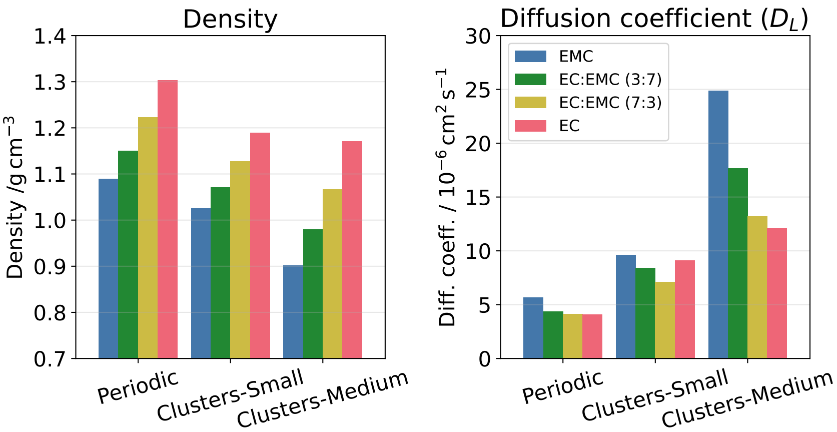

Figure S8: Densities (left) and diffusion coefficients (right) of different molecular composition after a 1 ns NPT simulation using models trained on different data sets, all labelled with `B97-D3`. The values of the `Cluster` models are averages over the different data set samples.

S4 Structural properties of liquids

This section contains RDFs evaluated over the various MD-trajectories considered in this work. Coordination numbers were also determined and tabulated in this section.

S4.1 Properties resulting from periodic data

RDFs of different liquid compositions are shown in Fig. S9. The RDFs are results of MD-simulations that employed MLIPs trained on periodic data labelled with different DFT-functionals. The RDFs were computed with respect to molecular centre-of-mass. The associated first-shell coordination numbers are listed in Table S8.

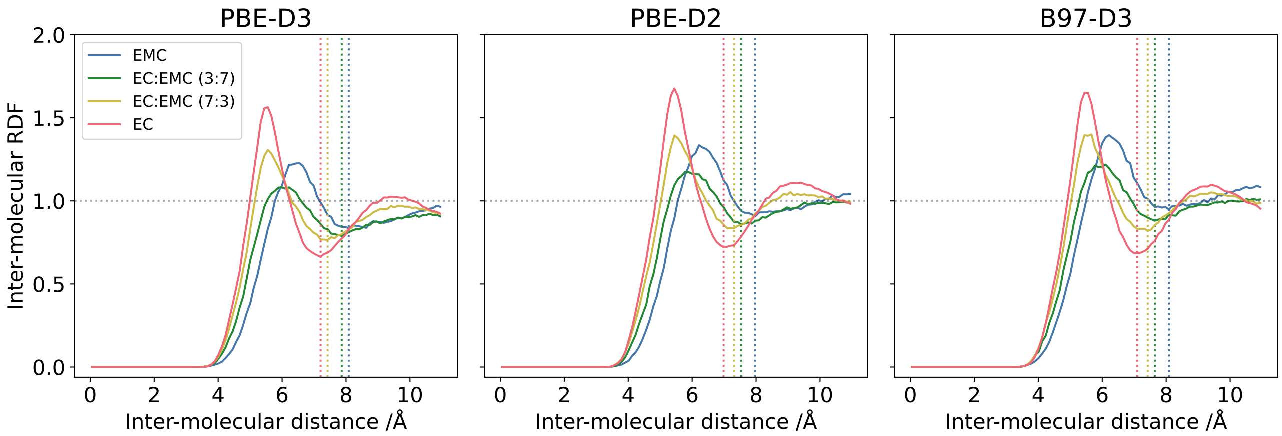

Figure S9: Inter-molecular RDFs over different molecular compositions (with 1000 atoms in each) over a 1 ns long NPT trajectory using models trained on periodic data that was labelled with different functionals. The vertical lines indicate the radius of the first solvation shell.

Table S8: Radius of first solvation shell (R), coordination number (CN) and average coordination number ($\overline{\text{CN}}$) associated with Fig. S9.

Functional	Composition	R	CN	$\overline{\text{CN}}$
PBE-D3	EMC	8.09	9.44	9.74
	EC:EMC (3:7)	7.86	9.85	
	EC:EMC (7:3)	7.43	9.45	
	EC	7.20	10.25	
PBE-D2	EMC	7.98	9.93	9.84
	EC:EMC (3:7)	7.54	9.36	
	EC:EMC (7:3)	7.32	9.82	
	EC	6.98	10.22	
B97-D3	EMC	8.09	10.87	10.42
	EC:EMC (3:7)	7.64	10.08	
	EC:EMC (7:3)	7.43	10.29	
	EC	7.10	10.43	

S4.2 Properties resulting from cluster data

RDFs of different liquid compositions are shown in Figs S10 and S11. The RDFs are results of MD-simulations that employed MLIPs trained on various cluster-based training sets with labels at the ωB97X-D3 level of theory. The MLIPs were trained with different training seeds and different versions of the training sets (which are discussed in the main paper). The RDFs were computed with respect to molecular centre-of-mass. The associated first-shell coordination numbers have been listed in Tables S9 and S10, respectively. Visual comparison of the coordination numbers are shown in Fig. S12.

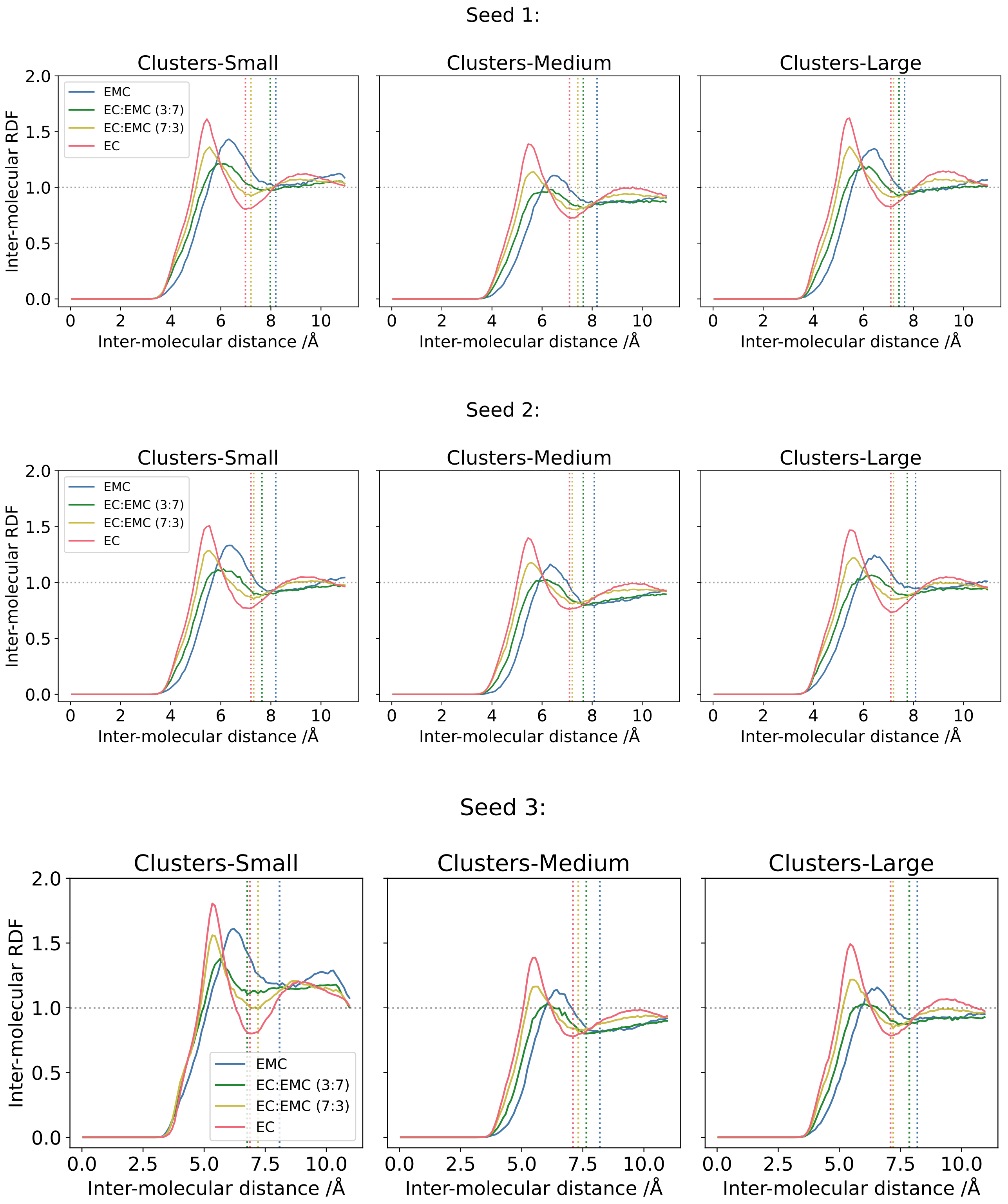

Figure S10: Inter-molecular RDFs over different molecular compositions (with 1000 atoms in each) over a 1 ns long NPT trajectory using models trained on cluster data that was labelled with ωB97X-D3. The vertical lines indicate the radius of the first solvation shell.

Table S9: Radius of first solvation shell (R), coordination number (CN) and average coordination number ($\overline{\text{CN}}$) associated with Fig. S10.

Seed 1:

Data set	Composition	R	CN	$\overline{\text{CN}}$
Cluster-Small	EMC	8.20	11.99	9.67
	EC:EMC (3:7)	7.98	12.31	
	EC:EMC (7:3)	7.20	9.83	
	EC	6.98	10.51	
Cluster-Medium	EMC	8.20	9.37	9.18
	EC:EMC (3:7)	7.64	8.54	
	EC:EMC (7:3)	7.43	9.14	
	EC	7.10	9.68	
Cluster-Large	EMC	7.64	8.76	11.16
	EC:EMC (3:7)	7.43	9.18	
	EC:EMC (7:3)	7.20	9.67	
	EC	7.10	11.07	

Seed 2:

Data set	Composition	R	CN	$\overline{\text{CN}}$
Cluster-Small	EMC	8.20	10.93	9.76
	EC:EMC (3:7)	7.64	9.69	
	EC:EMC (7:3)	7.32	9.58	
	EC	7.20	10.71	
Cluster-Medium	EMC	8.09	8.87	8.91
	EC:EMC (3:7)	7.64	8.71	
	EC:EMC (7:3)	7.20	8.42	
	EC	7.10	9.64	
Cluster-Large	EMC	8.09	10.06	10.23
	EC:EMC (3:7)	7.76	9.88	
	EC:EMC (7:3)	7.20	8.87	
	EC	7.10	10.22	

Seed 3:

Data set	Composition	R	CN	$\overline{\text{CN}}$
Cluster-Small	EMC	8.09	13.43	9.83
	EC:EMC (3:7)	6.76	7.58	
	EC:EMC (7:3)	7.20	10.74	
	EC	6.88	10.58	
Cluster-Medium	EMC	8.20	9.30	9.12
	EC:EMC (3:7)	7.64	8.65	
	EC:EMC (7:3)	7.32	8.85	
	EC	7.10	9.69	
Cluster-Large	EMC	8.20	9.97	10.58
	EC:EMC (3:7)	7.86	10.09	
	EC:EMC (7:3)	7.20	8.86	
	EC	7.10	10.41	

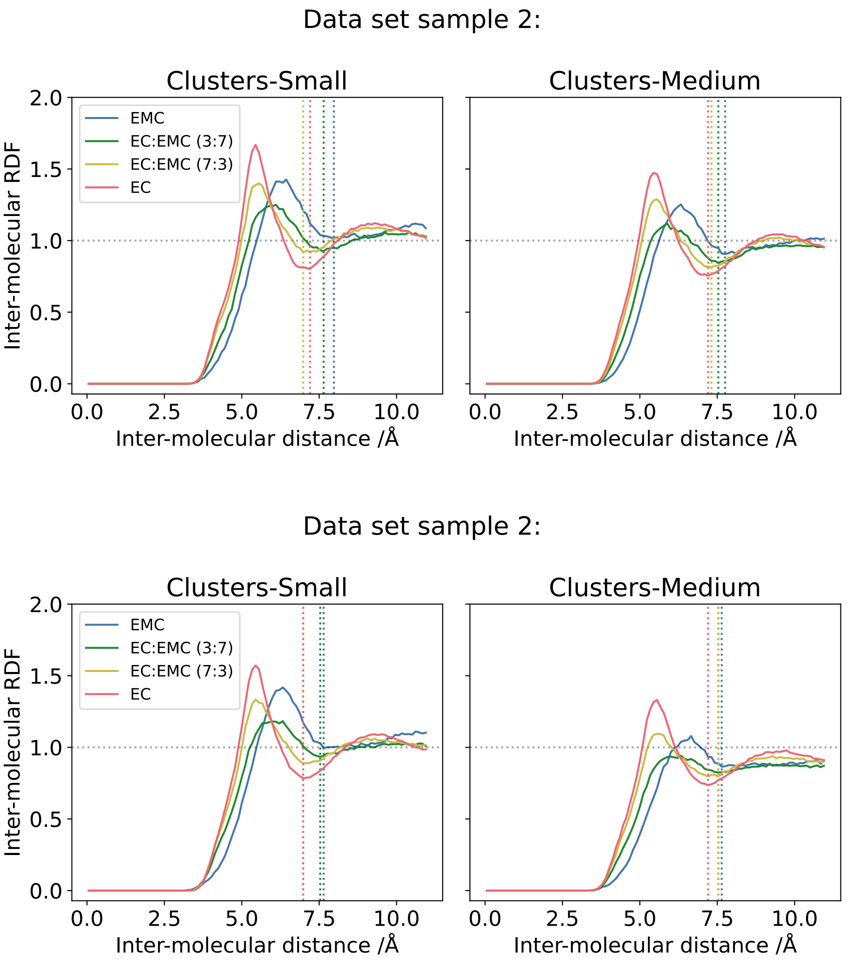

Figure S11: Inter-molecular RDFs over different molecular compositions (with 1000 atoms in each) over a 1 ns long NPT trajectory using models trained on cluster data that was labelled with ω`B97X-D3`. Differences between two different data set samples are shown in a and b, respectively. The first of the three data set samples is included in Fig. S10. The vertical lines indicate the radius of the first solvation shell.

Table S10: Radius of first solvation shell (R), coordination number (CN) and average coordination number ($\overline{\text{CN}}$) associated with Fig. S11.

Data set sample 2:

Data set	Composition	R	CN	$\overline{\text{CN}}$
Cluster-Small	EMC	7.98	10.87	9.41
	EC:EMC (3:7)	7.64	10.58	
	EC:EMC (7:3)	6.98	8.94	
	EC	7.20	11.54	
Cluster-Medium	EMC	7.76	8.64	10.48
	EC:EMC (3:7)	7.54	8.96	
	EC:EMC (7:3)	7.32	9.42	
	EC	7.20	10.60	

Data set sample 3:

Data set	Composition	R	CN	$\overline{\text{CN}}$
Cluster-Small	EMC	7.64	9.25	8.59
	EC:EMC (3:7)	7.54	9.82	
	EC:EMC (7:3)	6.98	8.57	
	EC	6.98	10.11	
Cluster-Medium	EMC	7.64	7.09	9.44
	EC:EMC (3:7)	7.54	7.94	
	EC:EMC (7:3)	7.54	9.45	
	EC	7.20	9.88	

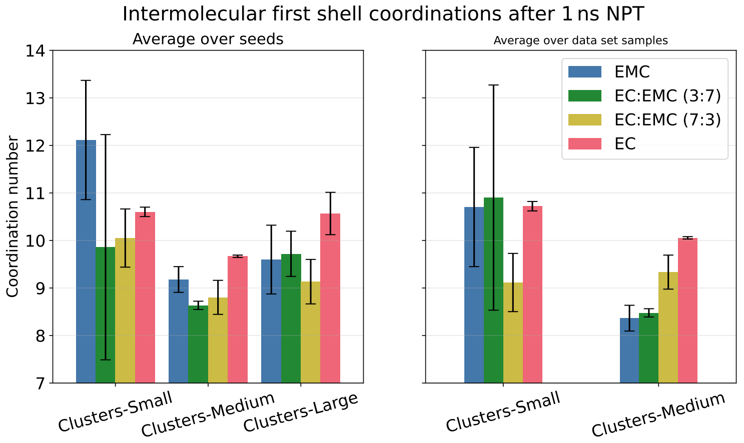

Figure S12: Average first shell coordinations pertaining to the different data set sizes where the error bars are the respective standard deviations. Averages were taken over all seeds (left) and data set samples (right).

References

[1] M. P. Allen and D. J. Tildesley. *Computer simulation of liquids.* Oxford University Press, Oxford, United Kingdom, second edition edition, 2017.

[2] Ioan-Bogdan Magdău, Daniel J Arismendi-Arrieta, Holly E Smith, Clare P Grey, Kersti Hermansson, and Gábor Csányi. Machine learning force fields for molecular liquids: Ethylene carbonate/ethyl methyl carbonate binary solvent. *npj Computational Materials*, 9(1):146, 2023.
